# MAGI3 Acts as an Intestinal Epithelial Gatekeeper in Inflammation‐Associated Colorectal Cancer by Suppressing the STAT3‐CCL2 Axis and TAM‐Mediated Immunosuppression

**DOI:** 10.1002/advs.78577

**Published:** 2026-10-31

**Authors:** Yumeng Yang, Zhigang Bai, Haibo Wang, Yu Guo, Yan Zhang, Meihan Hu, Shuaikang Fan, Ran Song, Tongmei Zhang, Mengqi Deng, Xiaomei Yang, Zhongtao Zhang, Junqi He

**Affiliations:** ^1^ Department of Biochemistry and Molecular Biology School of Basic Medical Sciences Capital Medical University Beijing China; ^2^ Laboratory For Clinical Medicine Capital Medical University Beijing China; ^3^ Department of General Surgery State Key Lab of Digestive Health Beijing Friendship Hospital Capital Medical University National Clinical Research Center for Digestive Diseases Beijing China; ^4^ Department of Medical Oncology Beijing Tuberculosis and Thoracic Tumor Research Institute Beijing Chest Hospital Capital Medical University Beijing China; ^5^ Beijing Obstetrics and Gynecology Hospital Capital Medical University Beijing Maternal and Child Health Care Hospital Beijing China

**Keywords:** CCL2, colorectal cancer, immunosuppression, MAGI3, STAT3, targeted therapy, tumor‐associated macrophages

## Abstract

Chronic intestinal inflammation is a major driver of colorectal cancer (CRC). As the mucosal barrier's frontline sentinel, the intestinal epithelium initiates the inflammation‐to‐cancer transition. However, the early cell‐intrinsic events that convert inflammatory responses into a sustained pro‐tumorigenic program remain unclear. In this study, we identify MAGI3 as an intestinal epithelial guardian progressively lost during inflammation‐associated colorectal tumorigenesis. Low MAGI3 correlates with heightened inflammatory activity and poor prognosis. Inflammation‐induced epithelial MAGI3 downregulation drives STAT3 hyperactivation, CCL2 upregulation, and recruitment of M2‐like tumor‐associated macrophages (TAMs), thereby promoting an immunosuppressive microenvironment and tumor progression. By contrast, single‐cell RNA sequencing (scRNA‐seq) demonstrated that MAGI3 overexpression reshapes the composition of TAMs and limits M2‐like TAM infiltration. Mechanistically, the PDZ1 domain of MAGI3 binds directly to the C‐terminus of STAT3, blocking the SH2 pocket, impairing the interaction between STAT3 and IL‐6R/JAK2, and ultimately inhibiting STAT3 phosphorylation at Tyr705. Clinically, the MAGI3‐STAT3 axis offers a new therapeutic strategy for chemotherapy‐resistant CRC, with low MAGI3 expression predicting enhanced sensitivity to STAT3 inhibitors. Collectively, these findings establish MAGI3 as a gatekeeper of the intestinal epithelium in inflammation‐associated CRC, with the MAGI3‐STAT3 axis regulating TAM recruitment. Targeting the MAGI3‐STAT3 axis could offer an early intervention to reverse inflammation‐to‐cancer transition.

## Graphical abstract

Our study identifies MAGI3 as an intestinal epithelial gatekeeper that restrains inflammation‐associated colorectal tumorigenesis but is itself suppressed under highly inflammatory conditions. MAGI3 deficiency activates the STAT3‐CCL2 signaling, thereby promoting TAM‐mediated immunosuppression and inflammation‐associated colorectal tumor progression. Targeting the MAGI3–STAT3 axis may provide a therapeutic strategy to interrupt the inflammation‐to‐cancer transition.

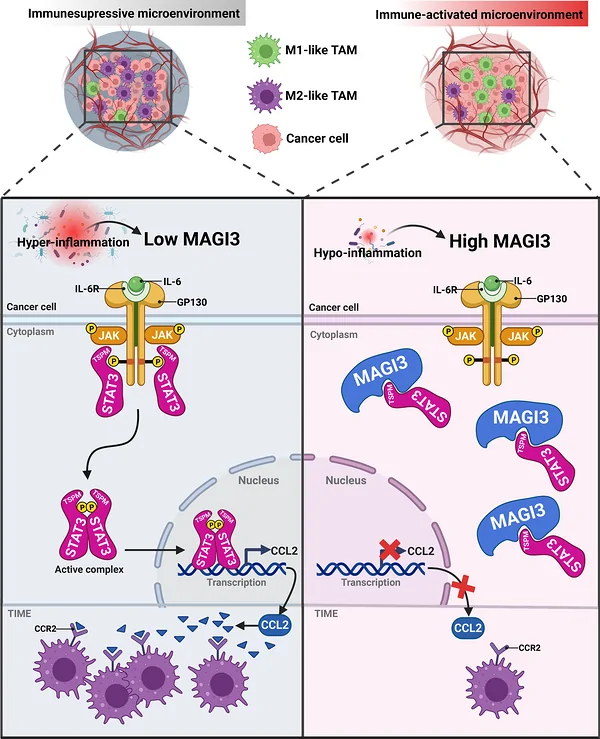

## 1 Introduction

Chronic inflammation is a well‐established driver of colorectal cancer (CRC) [1, 2]. It accounts for nearly all colitis‐associated cancers (CAC) and, in a substantial proportion of sporadic CRC, creates a pro‐tumorigenic niche through occult and persistent inflammatory insults [3, 4, 5]. Despite this well‐established epidemiological link, it remains unclear how an ordinarily self‐limited mucosal repair response is converted into a persistent, tumor‐promoting program in certain individuals [6]. A key knowledge gap is how epithelial‐intrinsic molecular “brakes” that restrain oncogenic signaling and promote inflammatory resolution in premalignant epithelium are progressively eroded during chronic inflammation, ultimately licensing malignant transformation.

Prior studies have largely focused on immune cell‐driven amplification of inflammation or on downstream consequences of pathway activation [7, 8]. However, intestinal epithelial cells (IECs) are not merely passive recipients. The IL‐6/JAK/STAT3 signaling axis constitutes a major molecular bridge linking epithelial inflammation to immune reprogramming [9, 10, 11]. Persistent STAT3 activation in IECs drives the production of key chemokines, including CCL2, which recruit monocytes and macrophages and instruct their polarization toward M2‐like tumor‐associated macrophages (TAMs), thereby establishing a self‐reinforcing pro‐tumorigenic microenvironment [12, 13]. This STAT3‐CCL2‐TAM axis serves as a critical amplifier of immunosuppression and tumorigenesis. Nevertheless, during the transition from colitis to cancer, the upstream mechanisms that govern STAT3 activation in IECs, and the manner in which this axis coordinates TAM recruitment and functional evolution, remain poorly defined [14, 15].

In parallel, during the inflammation‐to‐cancer transition, the tumor immune microenvironment (TME) progressively acquires pronounced immunosuppressive features [16, 17]. TAMs are central orchestrators of this process; their recruitment and polarization toward M2‐like, pro‐tumorigenic states are critically instructed by epithelial‐derived signals [18, 19, 20]. Importantly, TAM phenotypic and functional heterogeneity is profoundly shaped by the inflammatory milieu within the TME [19]. In established tumors, persistent inflammation often biases TAMs toward an immunosuppressive, M2‐like program characterized by enhanced IL‐10 and TGF‐β signaling [21], increased expression of markers such as CD163 and CD206, and augmented chemokine production. Clinically, enrichment of CD163^+^/CD206^+^ TAM subsets correlates with poor prognosis across multiple CRC cohorts [22]. Nevertheless, the functional heterogeneity, spatial organization, and dynamic reprogramming of TAMs within the TME underscore that single‐marker or static measurements cannot capture their true biology. Therefore, there is an urgent need to identify intestinal epithelial regulators of aberrant STAT3 activation that are perturbed early during inflammation and, over the course of the inflammation‐to‐cancer transition, link epithelial signaling to chemokine production and macrophage remodeling.

In this study, we analyzed five GEO datasets spanning normal mucosa, ulcerative colitis (UC), and colitis‐associated cancers (CAC) to identify differentially expressed genes (DEGs). We then applied the Mfuzz package for time‐course clustering to pinpoint genes exhibiting sustained, consistent changes across the normal‐inflammation‐tumor continuum, thereby systematically prioritizing candidate drivers of inflammation‐associated colorectal tumorigenesis. We identified membrane‐associated guanylate kinase, WW and PDZ domain‐containing protein 3 (*MAGI3*), a PDZ‐domain scaffold protein, as a critical intestinal epithelial gatekeeper that is downregulated early and progressively lost during inflammation‐associated colorectal tumorigenesis. Mechanistically, MAGI3 directly binds STAT3 via its PDZ1 domain, impairing JAK2‐STAT3 complex formation and consequently suppressing STAT3 phosphorylation and downstream CCL2 production. This epithelial‐intrinsic regulatory mechanism restrains the recruitment of immunosuppressive TAMs and limits tumor progression. Moreover, single‐cell RNA sequencing (scRNA‐seq) of orthotopic colorectal tumors revealed that *Magi3* overexpression selectively reduced STAT3 pathway activity and CCL2 expression in tumor epithelial cells. Pseudotime analysis further traced the dynamic evolution of TAM subpopulations during tumor progression in *Magi3*‐overexpressing versus control tumors, establishing a cell type‐specific link between epithelial signaling and the recruitment of M2‐like TAMs. Collectively, our findings establish MAGI3 within intestinal epithelial cells as a gatekeeper in the inflammation‐to‐cancer transition and suggest its potential as a predictive biomarker for guiding STAT3‐targeted therapy.

## 2 Materials and methods

### 2.1 Data Collection and CRC Samples

CRC genomic and clinical datasets were obtained from The Cancer Genome Atlas (TCGA) (RRID: SCR_003193) via the cBioPortal platform (https://www.cbioportal.org/). Public transcriptomic datasets were retrieved from the NCBI Gene Expression Omnibus (GEO) (RRID: SCR_005012), including GSE44988, GSE44904, GSE46200, GSE43338, GSE64658, GSE4183, GSE39582, GSE3629, GSE87466, GSE47908, GSE38713, GSE92415, GSE11223, GSE107499, GSE66407, GSE188711, and GSE72970. Proteomic data were accessed from the NCI Proteomic Data Commons (RRID: SCR_018273) (https://pdc.cancer.gov/pdc/). Data preprocessing for both TCGA and GEO datasets included quality control (QC) steps such as the removal of low‐quality samples, normalization of gene expression data, and log‐transformation for downstream analysis.

In addition, 230 primary CRC samples were collected from surgical resections performed at Beijing Friendship Hospital, Capital Medical University, between 2015 and 2018. All tissues were immediately snap‐frozen in liquid nitrogen or fixed in 4% paraformaldehyde for 24 h, followed by paraffin embedding. All procedures were approved by the institutional ethics committee (approval number: 2018‐P2‐045‐01), and informed consent was obtained from all patients.

### 2.2 Mouse Husbandry and Ethics

All mice were housed under specific pathogen‐free conditions in individually ventilated cages with a 12 h light/dark cycle, controlled temperature (22°C ± 1°C) and humidity (50 ± 10%), and provided with autoclaved food and water ad libitum. All animal experiments were performed in accordance with the NIH Guide for the Care and Use of Laboratory Animals and were approved by the Institutional Animal Care and Use Committee of Capital Medical University (protocol numbers: AEEI‐2020‐133 and AEEI‐2025‐112).

### 2.3 DSS and AOM‐DSS Models

*Magi3*‐knockout (*Magi3^−/−^*) mice were generated in collaboration with the Shanghai Model Organisms Center (Shanghai, China). Genotyping was conducted by PCR using the following primers. Wild‐type: 5'‐ATGCTTGCTTTCTTGAGTGTA‐3' (forward) and 5'‐GGCTTGGGTGTGCATAGAA‐3' (reverse), and knockout: 5'‐GAACACTTTCCCATGGTGCC‐3' (forward) and 5'‐CCAAACACAGGGCCAGGAAT‐3' (reverse). Mice were randomly assigned to experimental groups using a random number generator to ensure equal group sizes.

To induce chronic colitis, mice were administered DSS (36–50 kDa; Meilunbio, Cat# MB5535) in drinking water according to the following schedule: 2% DSS for 7 days, 1% DSS for 10 days, and 2% DSS for an additional 7 days.

For the AOM/DSS tumorigenesis model, male *Magi3^−/−^* (n = 5) and wild‐type (WT; n = 5) mice (5–6 weeks, 18–20 g) were injected intraperitoneally with azoxymethane (AOM; 9 mg/kg; Sigma‐Aldrich, Cat# A5486). After 7 days, mice underwent three cycles of DSS (36–50 kDa; Meilunbio, Cat# MB5535) treatment, with each cycle consisting of 2% DSS administration for 7 days followed by 14 days of regular drinking water. Body weight and clinical symptoms were monitored every three days. Animals were maintained on water until study endpoint.

At necropsy, the colon was excised, opened longitudinally, rinsed, rolled (“Swiss rolls”), fixed in 10% neutral buffered formalin for 24 h, paraffin‐embedded, and sectioned. Hematoxylin and eosin (H&E) ‐stained sections were histologically evaluated to assess tumor incidence, adenoma severity, and histopathological grade. Histological scoring was performed independently by two pathologists based on the presence and severity of ulceration, hyperplasia, and inflammatory infiltration, according to the criteria detailed in Table S1.

### 2.4 In Vivo Models

All mouse experiments were performed under NIH animal care guidelines and authorized by the Institutional Animal Care and Use Committee of Capital Medical University (protocol number AEEI‐2020‐133 and AEEI‐2025‐112). Mice were excluded only if they met predefined humane endpoints, and no animals were excluded for technical reasons. All histological and IHC assessments were conducted under blinded conditions.

A syngeneic tumor model was established by subcutaneously injecting MC38 cells (RRID: CVCL_B288) with or without MAGI3 overexpression into the flanks of 6‐week‐old male C57BL/6 mice (16–18 g, n = 4 per group) (RRID: IMSR_JAX:000664). Tumor growth was monitored; mice were euthanized on day 20 and harvested, weighed, and processed for immunohistochemistry (IHC) and flow cytometry.

For the macrophage depletion model, male BALB/c mice (16–18 g, n = 5 per group) (RRID: MGI:2683685) were intraperitoneally injected with 150 µl of clodronate liposomes or PBS liposomes as a control (Liposoma B.V., Netherlands, Cat# CP‐005‐005). The following day, CT26 cells (1 × 10^6^ cells/0.1 mL PBS) (RRID: CVCL_7254) were subcutaneously injected into mice. Subsequently, mice were administered 150 µl of clodronate liposomes or PBS liposomes intraperitoneally twice a week. Tumor volume was monitored every 3 days by caliper measurement. On day 16, mice were euthanized, and tumors were excised, fixed, paraffin‐embedded, and subjected to immunohistochemistry. Macrophage depletion was confirmed by F4/80 immunohistochemistry at the study endpoint.

For the Stattic (MCE, Cat# HY‐13818, Compound CID: 2779853) treatment study, CT26 cells (RRID: CVCL_7254) were implanted into male BALB/c mice (16‐18 g, n = 5 per group) (RRID: MGI:2683685). When tumors reached 50–100 mm^3^, mice received daily intraperitoneal injections of vehicle or Stattic (4 mg/kg). Tumor volume was monitored every 3 days by caliper measurement. Tumor volume was calculated using the formula V = (L × W^2^) / 2, where L and W represent the longest and shortest tumor diameters, respectively.

### 2.5 Orthotopic Cecal Tumor Model

Luciferase‐expressing CT26 cells (CT26‐Luc; 2 × 10^6^ cells in 25 µL) were mixed with 25 µL Matrigel (ABW, Cat# 0827335) and implanted into male BALB/c mice (16‐18 g, n = 3 per group; RRID: MGI:2683685). Mice were anesthetized with 2% isoflurane and administered buprenorphine intraperitoneally for preoperative analgesia (0.1 mg/kg; 100 µL). Anesthesia was maintained with 1.8% isoflurane. Following abdominal disinfection, a 10–15 mm midline incision was made, and the cecum was gently exteriorized onto sterile saline‐moistened gauze. The 50 µL cell suspension was injected into an avascular region of the cecal subserosa. After confirming the formation of a localized injection bleb without visible leakage or hemorrhage, the cecum was returned to the abdominal cavity, and the peritoneum and abdominal wall were closed separately. Mice were monitored until recovery and assessed regularly for body weight, activity, food intake, and wound healing.

Bioluminescence imaging was performed every 3 days after implantation. D‐luciferin potassium salt (IVISbrite D‐Luciferin Potassium Salt, PerkinElmer, Cat# 122799) was prepared at 30 mg/mL and administered intraperitoneally at 150 mg/kg body weight (5 µL/g). Images were acquired under isoflurane anesthesia at a consistent time point following D‐luciferin administration using a small‐animal imaging system. On day 19 post‐implantation, mice were euthanized, and the cecum and orthotopic tumors were collected. Tumor formation was documented, and tumor volume was measured.

### 2.6 Cell Culture and Authentication

All human CRC cell lines (HT29, HCT116, Caco‐2, RKO), human colon epithelial cell line (NCM460), and mouse cell lines (MC38, CT26, RAW264.7) were obtained from the American Type Culture Collection (ATCC) or the National Collection of Authenticated Cell Cultures (Chinese Academy of Sciences). Cells were cultured in DMEM or RPMI‐1640 medium supplemented with 10% fetal bovine serum (FBS) and 1% penicillin/streptomycin at 37°C in a 5% CO_2_ humidified atmosphere. Cell lines were authenticated using short tandem repeat (STR) profiling and routinely checked for mycoplasma contamination every 3 months to ensure their integrity. All experiments were performed with cells within 15 passages from thawing, and each key experiment was independently repeated at least three times unless otherwise specified.

### 2.7 Generation of *Magi3*‐knockout CT26 Cells

*Magi3*‐knockout CT26 cells were generated by CRISPR/Cas9‐mediated genome editing. Two sgRNAs flanking the target region of the mouse *Magi3* transcript NM_133853.3 were designed using CRISPOR based on predicted on‐target activity and specificity. sgRNA‐A1, located upstream of exon 7, had the sequence 5′‐CCTTAGTCTATCCTACCCAG‐3′ (PAM: AGG), whereas sgRNA‐A2, located downstream of exon 13, had the sequence 5′‐GCCACCACGAAACTTAAGGG‐3′ (PAM: GGG). The MIT specificity scores were 83 and 86, and the corresponding CFD specificity scores were 91 and 95, respectively.

Synthetic sgRNAs were complexed with recombinant Cas9 protein and electroporated into approximately 1 × 10^6^ logarithmically growing CT26 cells using a Neon Transfection System with a 100 µL format (Thermo Fisher Scientific, MPK5000) according to the manufacturer's instructions. Transient Cas9‐sgRNA ribonucleoprotein delivery was used to limit Cas9 exposure and reduce the likelihood of off‐target editing. Single‐cell clones were subsequently isolated and screened by PCR.

Genotyping was performed using three PCR assays with the following primers: Region 1‐F, 5′‐AATGGTTATTTGGTGATGAAACCT‐3′; Region 1‐R, 5′‐CCATCTTTCTAGCCCCAGGTC‐3′; Region 2‐F, 5′‐GGTTTTGAAACAGTGCCACAT‐3′; and Region 2‐R, 5′‐TTGCAGATTGTTTTCAGCAAGAT‐3′. Deletion‐junction PCR was performed using Region 1‐F and Region 2‐R. Homozygous deletion was identified by loss of the 977‐bp Region 1 and 804‐bp Region 2 wild‐type amplicons, together with detection of an approximately 649‐bp deletion‐junction product. PCR and Sanger sequencing confirmed an approximately 23.7‐kb deletion in clone 1G4, and loss of MAGI3 protein was verified by Western blotting. Clone 1B1, which underwent the same editing and clonal‐isolation procedures but retained the wild‐type *Magi3* locus, was used as a procedure‐matched negative clonal control. Clone 1G4 was used as the *Magi3*‐knockout line in subsequent experiments.

### 2.8 RNA Interference and Lentiviral Systems

Two independent siRNAs targeting MAGI3 (Sangon Biotech, Shanghai, China) were used: siMAGI3‐1, 5'‐GGUCCACCAUCAGGAACAAACUCAG‐3'; and siMAGI3‐2, 5'‐GAGAUGGACCUGACCAGUCUAUAUA‐3'. Cells were transfected with Lipofectamine LTX Plus reagent (Thermo Fisher Scientific, Cat# 15338100) per the manufacturer's instructions.

For stable knockdown, lentiviral particles encoding shRNAs against MAGI3 (Santa Cruz, Cat# sc‐42005‐V) were transduced into target cells at 1 × 10^6^ infectious units (IFU) per 200 µL. Non‐targeting shRNA was used as a control. Lentiviral vectors for MAGI3 overexpression (human *MAGI3* and murine *Magi3*; GeneChem, Shanghai) were used at 6 × 10^8^ TU/mL, with an MOI of 10 for 24 h. Transduced cells were selected with 2 µg/mL puromycin for 7–10 days.

### 2.9 RT‐qPCR Analysis

Subcutaneous syngeneic tumors were washed with PBS, and total RNA was extracted using TRIzol reagent (Invitrogen, Cat# 15596026). The RNA was then reverse transcribed into cDNA using PrimeScript reverse transcriptase (TaKaRa, Cat# 2690A). Quantitative real‐time PCR (qPCR) was performed using SYBR Green fluorescence‐based PCR master mix (Thermo Fisher, Cat# 4472919). All reactions were conducted in triplicate, with β‐actin as the internal reference gene. Primer sequences are listed in Table S2.

### 2.10 CCL2 Enzyme‐Linked Immunosorbent Assay

CCL2 protein levels in CT26 cell culture supernatants and mouse tumor tissues were quantified using a commercial mouse CCL2 ELISA kit (Shanghai Enzyme‐linked Biotechnology Co., Ltd., Cat# ml063804) according to the manufacturer's protocol. Culture supernatants from CT26 cells with *Magi3* overexpression or knockdown were collected and clarified by centrifugation to remove cells and debris. Orthotopic and subcutaneous tumor tissues were homogenized on ice, centrifuged, and the resulting soluble fractions were collected for analysis. Standards and appropriately diluted samples (100 µL per well) were added to the antibody‐precoated plate and incubated at 37°C for 1.5 h. After three washes, 100 µL of biotinylated detection antibody was added to each well and incubated at 37°C for 1 h. The plate was then washed four times, followed by incubation with 100 µL of streptavidin–horseradish peroxidase conjugate at 37°C for 30 min. After four additional washes, 50 µL each of chromogenic substrates A and B were added, and the reaction was developed at 37°C in the dark for 15 min. The reaction was terminated with 50 µL of stop solution, and absorbance at 450 nm was measured within 5 min using a microplate reader. CCL2 concentrations were calculated from the standard curve and adjusted for the corresponding sample dilution factors.

### 2.11 CCL2 Neutralization Treatment in the CT26 Syngeneic Tumor Model

A syngeneic subcutaneous tumor model was established by subcutaneously injecting 1 × 10^6^ CT26‐sgNC or CT26‐sgMAGI3 cells into BALB/c mice. When tumors reached approximately 100 mm3, mice were randomly allocated to four experimental groups: CT26‐sgNC with isotype control IgG, CT26‐sgNC with anti‐CCL2 antibody, CT26‐sgMAGI3 with isotype control IgG, and CT26‐sgMAGI3 with anti‐CCL2 antibody. The anti‐mouse CCL2 neutralizing antibody (Selleck, A2132, clone 2H5) was administered intraperitoneally at 10 mg/kg, three times per week, for a total of seven doses; the corresponding isotype‐matched IgG was administered using the same schedule.

Tumor volume was measured at the indicated time points as described above. At the experimental endpoint, 16 days after initiation of antibody treatment, mice were euthanized, and tumors were excised and weighed. Tumor tissues were subsequently fixed in 4% paraformaldehyde, embedded in paraffin, sectioned, and subjected to immunohistochemical staining as indicated.

### 2.12 Flow Cytometry

Tumors were digested with Liberase TM (50 µg/mL, Sigma, Cat# 5401119001) and DNase I (0.1 mg/mL, Sigma, Cat# DN25). Red blood cells (RBCs) were lysed with 0.17 m ammonium chloride (BD Biosciences, Cat# 555899). A total of 1 × 10^6^ cells were stained with: anti‐CD45‐BV650 (BioLegend, Cat# 103151, RRID: AB_2565884), anti‐F4/80‐PE (BioLegend, Cat# 123110, RRID: AB_893486), anti‐CD11b‐AF710 (Tonbo Biosciences, Cat# 80–0112, RRID: AB_2621979), and anti‐CD206‐APC (BioLegend, Cat# 141707, RRID: AB_10896057). Doublets were excluded based on using FSC‐A/FSC‐H and SSC‐A/SSC‐H gating strategies. Samples were acquired using a BD LSRFortessa flow cytometer with compensation performed using single‐color controls, and analyzed with FlowJo v10 software (version 10.8.1, RRID: SCR_008520).

### 2.13 Gene Set and Pathway Analyses

Gene Set Enrichment Analysis (GSEA, version 4.3.3) was performed on samples stratified by MAGI3 expression, using Hallmark gene sets and curated STAT3/MYC target collections. GSEA was performed using the “gene set” permutation type with 1000 permutations, and significance was defined as *FDR* < 0.25 and *p* < 0.05.

Differentially expressed genes were further analyzed by GO and KEGG (RRID: SCR_012773) enrichment using clusterProfiler (R package) (RRID: SCR_016884) [23], with *p* < 0.05 considered significant.

### 2.14 Protein Interaction Assays

Co‐immunoprecipitation (Co‐IP): Performed as previously described [24]. Briefly, cell lysates were incubated with the indicated antibodies and Protein A/G‐agarose beads. Immune complexes were washed and analyzed by SDS‐PAGE followed by Western blotting.

GST pull‐down assay [25]: GST‐STAT3 (wild‐type or ΔCT) fusion protein immobilized on glutathione‐Sepharose 4B beads was incubated with His‐tagged MAGI3 PDZ domains. Bound proteins were washed, eluted, and detected by immunoblotting using anti‐His (MBL, Cat# PM032, RRID: AB_3717676) antibodies. Competitive binding of MAGI3 and JAK2 for STAT3 was tested by adding recombinant His‐MAGI3‐PDZ1 to HT29 lysates. To evaluate the contribution of key PDZ1 residues, an F422A/I424A double mutant was generated by site‐directed mutagenesis, and the abilities of recombinant His–PDZ1‐WT and His–PDZ1‐F422A/I424A to bind STAT3 and interfere with the JAK2–STAT3 interaction were compared. Bead‐bound JAK2 and His‐tagged PDZ1 were detected by immunoblotting, whereas the amounts of GST fusion proteins and recombinant His–PDZ1 proteins were assessed by Coomassie blue staining.

All Co‐IP, pull‐down, and competitive binding assays were repeated independently three times with consistent results. Recombinant proteins were verified by SDS‐PAGE purity (> 95%).

### 2.15 Immunohistochemistry

Immunohistochemistry was carried out essentially as reported previously [25]. Briefly, Formalin‐fixed paraffin‐embedded (FFPE) sections were stained with primary antibodies: anti‐MAGI3 (Novus, NBP2‐17210, RRID: AB_3264055), anti‐F4/80 (Santa Cruz, Cat# sc‐52664, RRID: AB_629466), anti‐CD163 (CST, Cat# 93498, RRID: AB_2800204), anti‐CD206 (CST, Cat# 24595, RRID: AB_2892682), anti‐CCL2 (Santa Cruz, Cat# sc‐52701, RRID: AB_628867), and anti‐p‐STAT3 (Tyr705) (CST, Cat# 9145, RRID: AB_2491009). HRP‐linked secondary detection was used.

Staining intensity (0‐3) and proportion (0‐4) were independently scored by two blinded pathologists. H‐scores were calculated as intensity × proportion.

### 2.16 Single‐Cell Analysis

Single‐cell RNA sequencing (scRNA‐seq) libraries were prepared using the 10 × Genomics Chromium Single Cell 3' v3 Reagent Kit and sequenced on an Illumina NovaSeq 6000 at an average depth of 50,000 reads per cell. RAW scRNA‐seq data were processed and quality‐controlled using Seurat (version 5.2.1) [26, 27]. Quality control steps included the removal of empty droplets, doublets (identified using DoubletFinder and subsequently excluded), and low‐quality cells with fewer than 300 or more than 6,000 detected genes, > 15% mitochondrial counts, or < 3% of counts derived from ribosomal genes. Putative erythroid cells were identified based on elevated expression of hemoglobin genes, including Hba‐a1, Hba‐a2, Hbb‐bs, and Hbb‐bt, and were excluded following inspection of their expression profiles and clustering patterns. A total of six orthotopic tumor samples were analyzed, comprising three biological replicates from the control‐vector group and three from the *Magi3*‐overexpression group (n = 3 mice per group). After quality‐control filtering, 62,735 cells were retained, including 31,927 cells from control‐vector tumors and 30,808 cells from *Magi3*‐overexpressing tumors. To correct for batch effects between samples, the Canonical Correlation Analysis (CCA) algorithm implemented in Seurat was applied for data integration. Cell clustering was performed using the FindClusters function with 30 principal components (dims = 1:30) and a resolution parameter of 0.4.

STAT3 Signature Scoring: STAT3 pathway activity was estimated at the single‐cell level using the “IL6/JAK/STAT3 signaling” gene set from the MSigDB Hallmark gene sets. The enrichment score for each cell was calculated using the AddModuleScore function [28], which calculates the enrichment of the gene set for each cell.

Inflammatory Score: An inflammatory signature score was calculated for each cell using inflammation‐related gene sets from the MSigDB Hallmark gene sets. Similar to STAT3 signature scoring, the AddModuleScore function was used to quantify the relative enrichment of inflammatory gene expression programs in individual cells.

Trajectory inference: Monocyte‐macrophage lineage differentiation trajectories were reconstructed using Monocle 2 (v2.18.0) with DDRTree reduction [29, 30] based on the top 1,000 highly variable genes identified from the monocyte‐macrophage lineage. Cells were ordered in pseudotime and visualized with plot_cell_trajectory.

The code for single‐cell analysis, including data processing, STAT3 signature scoring, and trajectory inference, is available on GitHub (https://github.com/Fancy1123611/305Lab/tree/main).

### 2.17 Molecular Docking

Predicted protein structures were obtained from AlphaFold (STAT3: AF‐P40763‐F1‐v4; JAK2: AF‐O60674‐F1‐v4; MAGI3: AF‐Q5TCQ9‐F1‐v4). Protein‐protein docking was performed using the GRAMM server [31], and results were visualized and analyzed in PyMOL (RRID: SCR_000305). Hydrogen bonds were identified using the “find‐polar contacts between chains” function. High‐resolution figures were exported with: “ray 4000,4000; png image.png, width = 4000, height = 4000.”

### 2.18 Statistical Analysis

The sample size was determined by power analysis, using a significance level of α = 0.05 with 80% power to detect statistical differences. The power analysis was conducted using the G*Power software (version 3.1) to ensure adequate sample size for detecting the expected effect size. Statistical analyses were conducted using R (version 4.4.1), SPSS (version 26) (RRID: SCR_002865), and GraphPad Prism (version 10.0) (RRID: SCR_002798).

Normality of the data was assessed using the Shapiro‐Wilk test (n ≤ 50) or Kolmogorov‐Smirnov test (n > 50). Normally distributed variables were compared using a two‐tailed unpaired Student's t‐test; non‐parametric data were analyzed using the Mann‐Whitney U test. For multiple group comparisons, one‐way or two‐way ANOVA was performed with the appropriate post‐hoc tests (Tukey's or Šidák's), as indicated in the figure legends. Outliers were identified using the Grubbs test for normally distributed data. Data points identified as outliers were excluded if they significantly affected the analysis (*p* < 0.05).

Categorical data were analyzed using the Chi‐square or Fisher's exact test, depending on sample size. Survival analysis was performed using the Kaplan‐Meier method, and statistical significance was assessed with the log‐rank test. Correlation analysis was performed using Pearson's or Spearman's correlation coefficients, depending on the data distribution. A significance threshold of *p* < 0.05 was applied for all tests.

## 3 Results

### 3.1 Inflammation‐Driven Stepwise Downregulation of Intestinal Epithelial MAGI3 From Normal Mucosa to Cancer Correlates with Inflammatory Activity and Poor Prognosis

To explore how inflammation drives CRC, we performed differential expression analyses of transcriptomic datasets from UC and CAC patients (GSE3629, GSE87466, GSE47908, GSE10616, and GSE38713) from two complementary comparisons—normal mucosa versus colitis and colitis versus cancer—to identify core genes implicated in the inflammation‐to‐cancer transition. In total, 787 DEGs were identified (Figure 1A). To further identify potential regulatory genes that are persistently downregulated or upregulated during the transition from chronic inflammation to tumorigenesis, we integrated three public datasets (GSE3629, GSE37283, and GSE47908) and corrected for batch effects (Figure S1A‐D). We then applied soft clustering (fuzzy c‐means) using the Mfuzz R package to 787 DEGs to delineate expression clusters exhibiting consistent trajectories across normal mucosa, UC, and CAC in the integrated dataset. The clustering analysis revealed that clusters 2, 3, 4, 8, 11, and 12 exhibited gradual and consistent expression changes along disease progression from normal tissue to UC and then to CAC, suggesting potential involvement in inflammation‐driven tumor development (Figure 1B). Next, we intersected the 396 genes from these six clusters with 765 TCGA‐defined prognostic genes significantly associated with patient survival, identifying 15 candidate genes (Figure 1C). Subsequently, we interrogated the expression of the 15 candidate genes in the scRNA‐seq dataset GSE231559 of primary CRC lesions. Five genes, including MAGI3, TEX11, SPINT1, CXADR, and ZDHHC11, displayed predominant expression within epithelial cells (Figure S1E,F), while the remaining genes exhibited more dispersed expression patterns, suggesting that these five genes may represent potential regulators of tumor epithelial biology. Importantly, a random forest model identified MAGI3 as the top‐ranked feature among these five epithelial‐expressed genes, with the highest feature importance score (Figure 1D). Based on its epithelial‐enriched expression pattern and top ranking in the machine‐learning model, we further focused on MAGI3 and quantitatively assessed its distribution across different cell populations in the scRNA‐seq dataset GSE231559. MAGI3‐expressing cells were predominantly detected in the epithelial population, accounting for 17.0% of epithelial cells, a proportion markedly higher than those observed in T cells, myeloid cells, B cells, endothelial cells, and plasma cells (Figure S1G). The expression profiles of canonical cell‐type markers further supported the reliability of the cell annotations and showed that MAGI3 expression was predominantly enriched in epithelial cells, with minimal expression detected in immune, stromal, or endothelial cell populations (Figure S1H).

**FIGURE 1:**
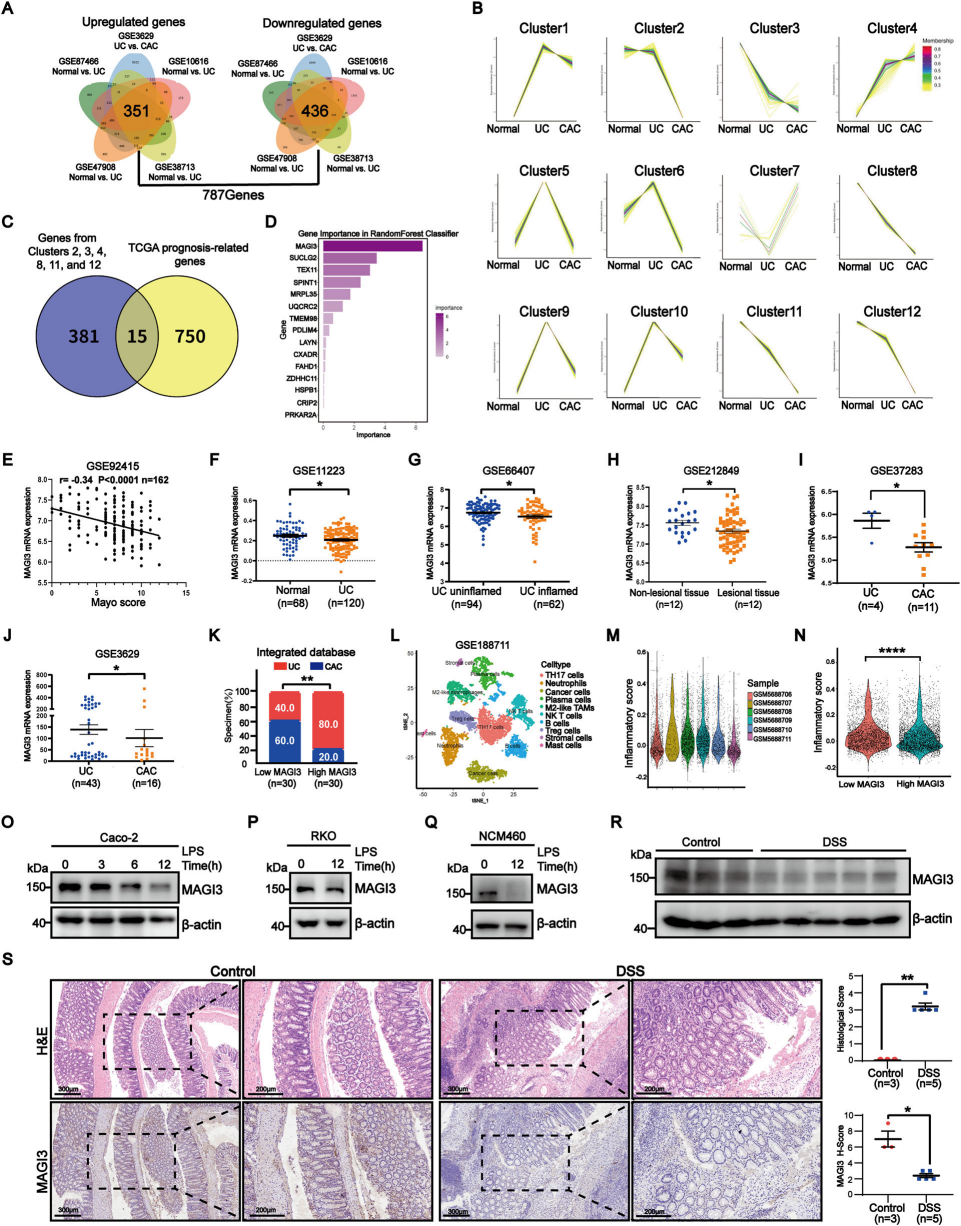
MAGI3 is progressively lost during the transition from normal mucosa to inflammation and inflammation‐associated CRC, and this sustained loss is closely linked to tumorigenesis. **A**. Identification of 787 differentially expressed genes (DEGs) common to ulcerative colitis (UC) and colitis‐associated cancer (CAC) across five transcriptomic datasets. DEGs were defined by |log_2_FC| > 0.3 and adjusted p < 0.05. **B**. Temporal expression patterns of DEG clusters during the normal‐UC‐CAC sequence, identified by Mfuzz soft clustering (v2.56.0). **C**. Venn diagram illustrating 15 risk genes shared between our clustered gene set (n = 396) and prognosis‐related genes from the TCGA cohort (n = 765). **D**. Bar plot showing the 15 genes contributing to tumor risk scores in the random forest model in GSE9348. **E**. Negative correlation between MAGI3 mRNA levels and Mayo endoscopic score in UC patients from the GSE92415 cohort(Pearson correlation, n = 162, r = ‐0.34, p < 0.05). **F**. MAGI3 mRNA is downregulated in UC patients compared to normal controls from the GSE11223 cohort (two‐tailed unpaired Student's t‐test, n = 68 for normal, n = 120 for UC). **G**. MAGI3 mRNA is reduced in inflamed versus non‐inflamed UC mucosa from the GSE66407 cohort (two‐tailed unpaired Student's t‐test, n = 94 for UC uninflamed, n = 62 for UC inflamed). **H**. MAGI3 expression is further reduced in actively lesioned areas compared with non‐lesioned areas of UC patient tissues from the GSE212849 cohort (Mann‐Whitney U test, n = 12 for non‐lesional tissue, n = 12 for lesional tissue). **I**. MAGI3 mRNA is decreased in CAC versus UC from the GSE37283 cohort (Mann‐Whitney U test, n = 5 for UC, n = 11 for CAC). **J**. MAGI3 mRNA is lower in CAC than in UC tissues from the GSE3629 cohort (Mann‐Whitney U test, n = 43 for UC, n = 16 for CAC). **K**. CAC patients show a higher frequency of low MAGI3 expression (Fisher's exact test, n = 30 for UC, n = 30 for CAC). **L‐N**. Single‐cell transcriptomic analysis of colon tissues from patients with varying MAGI3 expression levels from the GSE188711 dataset: t‐SNE plot of major cell types **(L)**, distribution of inflammatory scores across samples **(M)**, significantly elevated inflammatory scores in MAGI3‐low epithelial cells (**N**, Wilcoxon rank‐sum test). **O‐Q**. LPS stimulation (100 ng/mL; 3, 6, or 12 h) reduces MAGI3 protein levels in Caco‐2 **(O)**, RKO **(P)**, and NCM460 **(Q)** cells. n = 3 independent biological replicates. **R‐S**. DSS‐induced chronic colitis in mice: Western blot of MAGI3 in colon tissues **(R)**, representative H&E and IHC images (left, **S**), histological scores (upper right, S; Mann‐Whitney U test), and MAGI3 IHC quantification (lower right, S; Mann‐Whitney U test). For Western blot, histological scores, and IHC quantification, n = 3 mice/group for Control, n = 5 mice/group for DSS. All quantitative data are presented as mean ± SEM. ^*^ *p* < 0.05, ^**^ *p* < 0.01, ^***^ *p* < 0.001, ^****^ *p < 0.0001; ns, not significant*.

To validate this cell type‐specific distribution, we analyzed two independent CRC scRNA‐seq datasets, GSE132465 and GSE221575, both of which showed a consistent epithelial‐predominant expression pattern of MAGI3. In GSE132465, MAGI3 expression was mainly enriched in epithelial cells, with MAGI3‐expressing cells accounting for 17.2% of the epithelial population, compared with only 0.3%‐1.3% of cells in B‐cell, stromal, mast cell, T cell, and myeloid populations (Figure S2A–D). Similarly, in GSE221575, MAGI3‐expressing cells accounted for 27.8% of epithelial cells, markedly higher than the corresponding proportions in myeloid cells, T cells, fibroblasts, endothelial cells, and B cells, which ranged from 0.2% to 1.9% (Figure S2E–H). Collectively, three independent CRC scRNA‐seq datasets consistently demonstrated preferential epithelial expression of MAGI3 in colorectal tumors.

Having established the epithelial‐enriched expression pattern of MAGI3 across multiple CRC single‐cell RNA sequencing datasets, we next investigated whether MAGI3 expression was associated with intestinal inflammation and the progression from UC to CAC. MAGI3 levels were inversely correlated with inflammatory disease activity (Mayo score) in patients with UC (r = ‐0.34; Figure 1E). Compared with normal tissues, MAGI3 expression was downregulated in UC (Figure 1F, Figure S3A) and was markedly reduced in inflamed regions (Figure 1G) as well as ulcerated lesions (Figure 1H, Figure S3B). Relative to UC tissues, MAGI3 expression was further decreased in CAC (Figures 1I,J), and patients with low MAGI3 showed a higher prevalence of CAC (Figure 1K). Subsequently, we further examined the relationship between MAGI3 expression and inflammation in the GSE188711 scRNA‐seq dataset and found that patients with low MAGI3 expression in the cancer cell subpopulation exhibited higher inflammatory scores (Figures 1L–N, Figure S3C).

To determine whether inflammation is a direct driver of epithelial MAGI3 downregulation, we stimulated the CRC cell lines Caco‐2 and RKO with lipopolysaccharide (LPS), as well as the normal intestinal epithelial cell line NCM460, with LPS and assessed MAGI3 expression. The results showed that after LPS treatment, MAGI3 protein levels were significantly decreased in both CRC cell lines Caco‐2 and RKO (Figures 1O,P), and in normal intestinal epithelial cell line NCM460 (Figure 1Q).

We next established a DSS‐induced colitis model and evaluated MAGI3 expression in colonic tissues (Figure S4A). Compared with control mice, DSS‐treated mice displayed shortened colon length (Figure S4B,C), increased spleen index (Figure S4D), elevated disease activity index (Figure S4E), marked body‐weight loss (Figure S4F), and aggravated preneoplastic lesions (Figure S4G), indicating successful induction of colonic inflammatory injury. Consistently, both western blotting and immunohistochemistry revealed a pronounced decrease in MAGI3 protein levels in colonic tissues from DSS‐treated mice (Figures 1R,S). These findings were further corroborated by independent GEO datasets from AOM/DSS‐induced mouse models of CAC, in which MAGI3 expression was likewise downregulated in inflammation‐associated tumor tissues (Figure S5A–E).

Collectively, these results indicate that MAGI3 exhibits a pronounced epithelial cell‐ enriched expression pattern in colorectal tumors and is progressively downregulated along the inflammation‐to‐cancer continuum. Low MAGI3 expression is closely associated with heightened inflammatory activity and unfavorable clinical outcomes in inflammation‐associated CRC, suggesting that loss of epithelial MAGI3 may contribute to the inflammation‐to‐cancer transition.

### 3.2 MAGI3 Deficiency Promotes Inflammation‐Driven Colorectal Carcinogenesis via CCL2‐Mediated Macrophage Recruitment and M2‐Like TAM Polarization

To investigate the role of *Magi3* in the intestinal inflammation‐to‐cancer transition, we generated *Magi3*‐knockout mice. We first performed a systematic physiological assessment of *Magi3^−^* ^/−^ mice to determine whether *Magi3* deficiency affected general growth and development or the structure and function of major organs, particularly the intestine (Figure S6A–C). Compared with WT mice, *Magi3^−/−^* mice showed no significant differences in body weight (Figure S6D), colon length (Figure S6E), or spleen index (Figure S6F), and histological examination of major organs revealed no apparent abnormalities (Figure S6G), indicating that MAGI3 loss alone is insufficient to induce spontaneous intestinal inflammation or tissue injury under basal conditions. We therefore employed the AOM/DSS‐induced CAC model to investigate the role of MAGI3 deficiency in inflammation‐driven tumorigenesis (Figure 2A). Compared with wild‐type controls, *Magi3*‐knockout mice exhibited more severe disease phenotypes (Figure 2B), including increased tumor number and size (Figures 2C–E) and showed a higher incidence of high‐grade dysplasia (Figures 2F,G).

**FIGURE 2:**
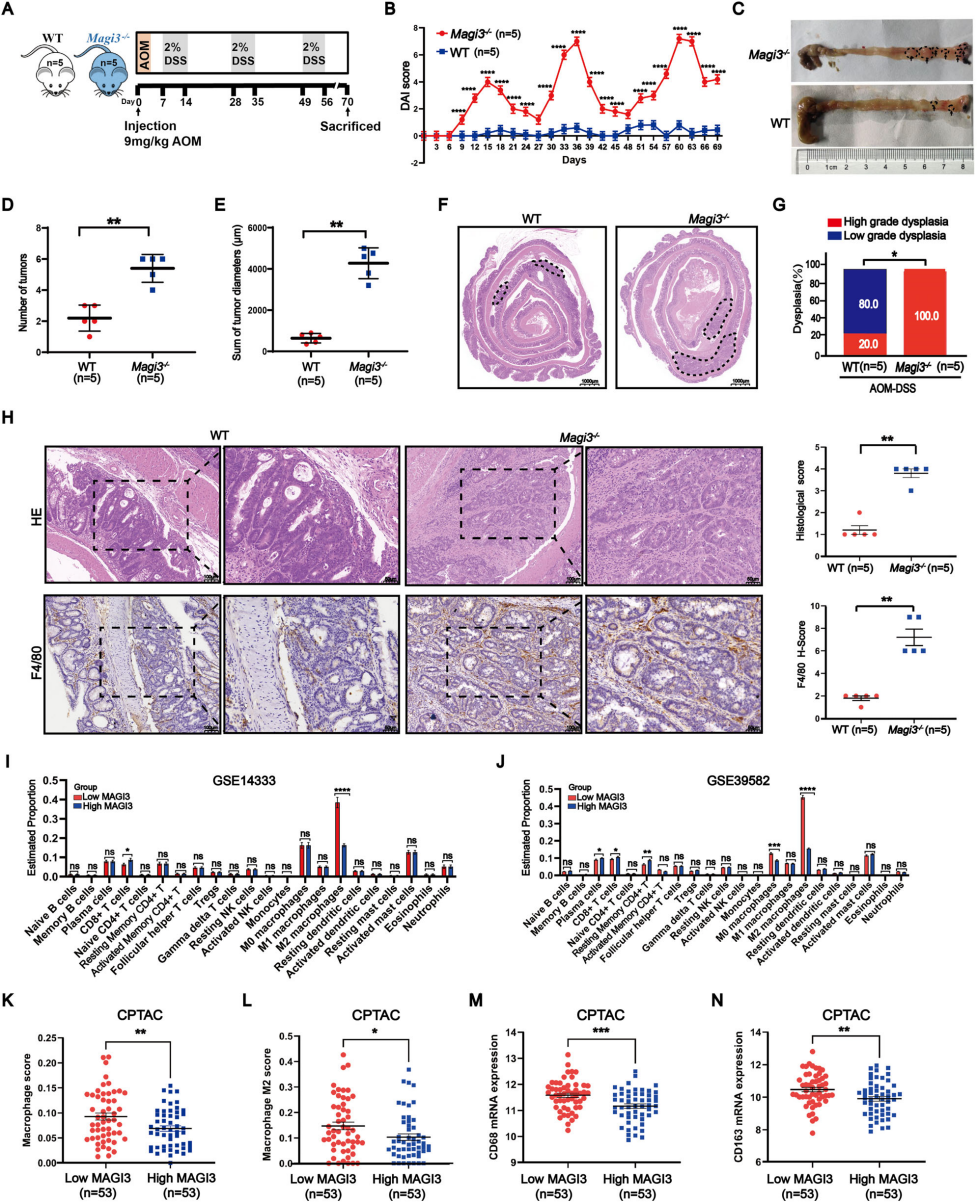
Loss of epithelial MAGI3 promotes inflammation‐associated colorectal tumorigenesis and macrophage infiltration. **A**. Schematic of the AOM/DSS‐induced colitis‐associated cancer (CAC) model in *Magi3^−/−^* and wild‐type (WT) mice. n = 5 mice/WT group, n = 5 mice/*Magi3^−/−^* group. **B**. Disease activity index (DAI) scores over time (two‐way ANOVA with Šidák's multiple comparisons test; n = 5 mice/group). **C**. Representative colon images. **D‐E**. Tumor number **(D)** and tumor load **(E)** at endpoint (Mann‐Whitney U test; n = 5 mice/group). **F**. H&E staining of Swiss‐roll sections. **G**. Proportion of mice with low‐grade dysplasia (LGD) or high‐grade dysplasia (HGD) (Fisher's exact test; n = 5 mice/group). **H**. Representative H&E and F4/80 immunohistochemistry (IHC) images (left), histological scores (upper right, Mann‐Whitney U test; n = 5 mice/group) and quantification of F4/80 IHC staining intensity (lower right, Mann‐Whitney U test; n = 5 mice/group). **I‐J**. Immune cell infiltration profiles stratified by MAGI3 expression in the GSE14333 **(I)** and GSE39582 **(J)** CRC cohorts. Patients were classified into MAGI3‐low and MAGI3‐high groups (GSE14333: n = 62 per group; GSE39582: n = 292 per group), and immune cell proportions were estimated using the CIBERSORT algorithm (two‐tailed unpaired Student's t‐test). **K**. Macrophage enrichment scores were estimated from CPTAC CRC RNA‐seq data using xCell in tumors stratified according to the median MAGI3 mRNA expression level (two‐tailed unpaired Student's t‐test). n = 53 patients per group. **L**. M2‐like macrophage scores derived from CIBERSORT‐based deconvolution of CPTAC CRC RNA‐seq data in tumors stratified into MAGI3‐high and MAGI3‐low groups according to MAGI3 mRNA expression (Mann‐Whitney U test). n = 53 patients per group. **M‐N**. The mRNA levels of CD68 **(M)** and CD163 **(N)** were decreased from low MAGI3 to high MAGI3 in CPTAC (two‐tailed unpaired Student's t‐test). n = 53 patients for each group. All quantitative data are presented as mean ± SEM. ^*^ *p* < 0.05, ^**^ *p* < 0.01, ^***^ *p* < 0.001, ^****^ *p < 0.0001; ns, not significant*.

We next examined whether the enhanced tumorigenesis caused by *Magi3* loss was accompanied by remodeling of the tumor immune microenvironment. Immunohistochemical analysis of multiple immune cell populations revealed markedly increased infiltration of F4/80^+^ macrophages in intestinal tumors from *Magi3^−/−^* mice (Figure 2H), with a concomitant modest decrease in CD8^+^ T‐cell infiltration. In contrast, no consistent significant differences were observed in NK cells, Tregs, CD4^+^ T cells, or B cells (Figure S7). These findings indicate that *Magi3* loss preferentially alters the macrophage compartment and suggest that macrophages may contribute to the tumor‐promoting effects of *Magi3* deficiency.

To determine whether this macrophage‐associated immune phenotype was clinically relevant, we applied the CIBERSORT algorithm to estimate the relative abundance of 22 immune cell subsets in two independent CRC transcriptomic cohorts, GSE14333 and GSE39582. In both cohorts, the most prominent and reproducible alteration associated with low MAGI3 expression was an increased proportion of M2‐like macrophages, accompanied by a modest reduction in the proportion of CD8^+^ T cells (Figures 2I,J). Plasma cells and resting memory CD4^+^ T cells were also slightly reduced in GSE39582; however, these changes were smaller than the increase in M2‐like macrophages and were not consistently observed across both cohorts.

We further validated the association between MAGI3 expression and macrophage infiltration using RNA‐seq data from the CPTAC CRC cohort. Tumors were stratified into MAGI3‐high and MAGI3‐low groups according to the median MAGI3 mRNA expression level. Compared with the MAGI3‐high group, tumors in the MAGI3‐low group exhibited a significantly higher xCell‐derived macrophage enrichment score (Figure 2K). CIBERSORT‐based immune cell deconvolution analysis of the same RNA‐seq dataset further revealed a significantly higher M2‐like macrophage score in the MAGI3‐low group (Figure 2L). Consistently, the pan‐macrophage marker CD68 and the M2‐like macrophage marker CD163 were markedly upregulated in MAGI3‐low tumors (Figures 2M,N). Collectively, these findings identify macrophage remodeling, particularly M2‐like TAM accumulation, as the predominant immune alteration associated with MAGI3 loss.

Given the established contribution of TAMs to the inflammation‐to‐cancer transition [32, 33], we next investigated whether tumor cell‐intrinsic MAGI3 regulates macrophage recruitment. In vitro Transwell co‐culture assays using conditioned medium derived from control and *Magi3*‐knockdown CT26 cells to evaluate RAW264.7 macrophage migration further confirmed that MAGI3 knockdown significantly enhanced macrophage migratory capacity (Figures 3A,B).

**FIGURE 3:**
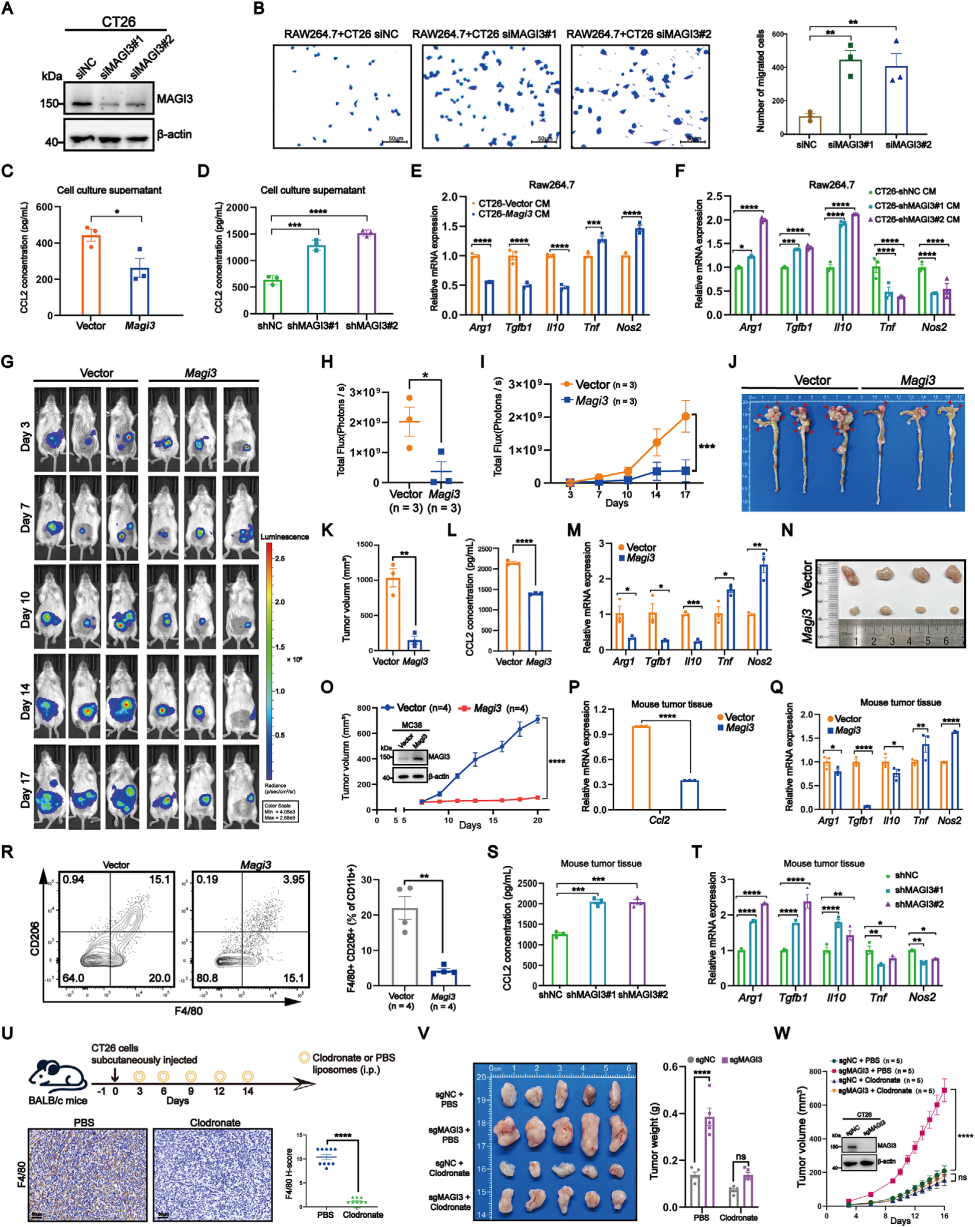
MAGI3 restrains CCL2‐mediated macrophage recruitment and M2‐like TAM polarization to suppress tumor growth. **A**. Western blot analysis confirmed successful and efficient transient transfection of CT26 cells with two independent siRNA sequences targeting MAGI3 (siMAGI3#1 and siMAGI3#2), compared to non‐targeting control (siNC). n = 3 independent biological replicates. **B**. Representative images (**left**; scale bars, 50 µm) and quantification (**right**) of RAW264.7 cells migrated toward conditioned media from CT26‐siNC, CT26‐siMAGI3#1, or CT26‐siMAGI3#2 cells in a Transwell assay. Migrated cells were quantified within 200 µm fields of view. n = 3 independent biological replicates; statistical significance was determined using one‐way ANOVA with Tukey's multiple‐comparisons test. **C–D**. ELISA analysis of CCL2 levels in culture supernatants from CT26 cells with *Magi3* overexpression or knockdown (**C**: n = 3 independent biological replicates, two‐tailed unpaired Student's *t*‐test; **D**: n = 3 independent biological replicates, one‐way ANOVA followed by Tukey's multiple‐comparisons test). **E–F**. qPCR analysis of M2‐associated markers (*Arg1, Tgfb1*, and *Il10*) and M1‐associated markers (*Tnf* and *Nos2*) in RAW264.7 cells cultured with conditioned medium from CT26 cells with MAGI3 overexpression or knockdown (n = 3 independent biological replicates; two‐tailed unpaired Student's t‐test). **G–I**. In vivo bioluminescence imaging of orthotopic cecal tumors established with vector‐control or *Magi3*‐overexpressing cells. Representative bioluminescence images **(G)**, endpoint total photon flux **(H)**, and longitudinal changes in total photon flux **(I)** are shown (n = 3 mice per group; **H**: two‐tailed unpaired Student's t‐test; **I**: two‐way ANOVA followed by Šidák's multiple‐comparisons test). **J–M**. Representative images of orthotopic tumors **(J)**, tumor‐volume quantification **(K)**, and ELISA analysis of CCL2 protein expression levels **(L)** and qPCR analysis of M2‐associated markers (*Arg1, Tgfb1*, and *Il10*) and M1‐associated markers (*Tnf* and *Nos2*) in tumor tissues **(M)** (n = 3 mice per group; two‐tailed unpaired Student's t‐test). **N‐O**. Representative images of excised tumors **(N)** and tumor‐growth curves **(O)** in the MC38 syngeneic model established with vector‐control or *Magi3*‐overexpressing cells. Inset: immunoblot confirmation of MAGI3 expression (n = 4 mice per group; two‐way ANOVA followed by Šidák's multiple‐comparisons test). **P‐Q**. qPCR analysis of *Ccl2* **(P)** and macrophage polarization‐associated genes **(Q)** in MC38 tumor tissues from the vector‐control and *Magi3*‐overexpression groups (n = 3 mice per group; two‐tailed unpaired Student's *t*‐test). **R**. Flow cytometric analysis of CD11b^+^F4/80^+^CD206^+^ M2‐like macrophages in MC38 tumors with or without *Magi3* overexpression (n = 4 mice per group; two‐tailed unpaired Student's *t*‐test). **S‐T**. ELISA quantification of CCL2 protein levels **(S)** and qPCR analysis of M2‐associated markers (*Arg1*, *Tgfb1*, and *Il10*) and M1‐associated markers (*Tnf* and *Nos2*) **(T)** in CT26 tumor tissues from the indicated control and *Magi3*‐knockdown groups (n = 3 mice per group; one‐way ANOVA followed by Tukey's multiple‐comparisons test). **U**. Schematic of the macrophage‐depletion experiment in the CT26 syngeneic tumor model **(top)**, with representative F4/80 immunohistochemical staining and corresponding H‐score quantification (**bottom**; two‐tailed unpaired Student's *t*‐test). **V‐W**. Effects of macrophage depletion on MAGI3 deficiency‐driven tumor growth. Representative images of excised tumors and tumor‐weight quantification **(V)** and tumor‐growth curves **(W)** from mice bearing CT26‐sgNC or CT26‐sgMAGI3 tumors treated with PBS or clodronate liposomes. Inset: immunoblot analysis of MAGI3 expression in sgNC and sgMAGI3 cells (n = 5 mice per group; **V, right**: two‐tailed unpaired Student's *t*‐test; **W**: two‐way ANOVA followed by Šidák's multiple‐comparisons test). All quantitative data are presented as mean ± SEM. ^*^ *p* < 0.05, ^**^ *p* < 0.01, ^***^ *p* < 0.001, ^****^ *p < 0.0001; ns, not significant*.

Given the critical role of chemokines in TAM recruitment [34], we conducted chemokine profiling to investigate the correlation between MAGI3 expression and various chemokines (Figure S8A). The results showed that MAGI3 was significantly negatively correlated with CCL2, CCL5, CCL8, CXCL9, and CXCL10. However, analysis of an independent dataset further revealed a negative correlation between MAGI3 and CCL2, whereas no significant associations were observed with CCL5, CCL8, CXCL9, and CXCL10 (Figure S8B–F). Multiple additional independent datasets consistently validated a strong inverse correlation between MAGI3 and CCL2 (Figure S8G–N), suggesting that MAGI3 preferentially impacts CCL2 expression rather than other chemokines.

ELISA further demonstrated that MAGI3 overexpression significantly reduced secreted CCL2 levels in the conditioned medium (Figure 3C), whereas MAGI3 knockdown markedly increased them (Figure 3D). Meanwhile, analysis of the polarization status of RAW264.7 cells cultured with CT26‐conditioned medium showed that, under conditions of MAGI3 overexpression, the M2‐associated markers *Arg1*, *Tgfb1*, and *Il10* were markedly downregulated (Figure 3E), whereas the M1‐associated markers *Tnf* and *Nos2* were upregulated; conversely, MAGI3 deficiency induced the opposite pattern of changes (Figure 3F). Collectively, these in vitro findings suggest that MAGI3 suppresses CCL2 secretion, thereby limiting macrophage migration and polarization toward an M2‐like phenotype.

To further investigate the role of MAGI3 in regulating tumor growth and TAM phenotypes in vivo, we established a mouse orthotopic cecal implantation model. In vivo bioluminescence imaging showed that MAGI3 overexpression markedly reduced orthotopic tumor burden (Figure 3G), as evidenced by significant reductions in both total photon flux (Figure 3H) and diminished bioluminescent signals over time (Figure 3I) and tumor volume (Figures 3J,K) on day 17. ELISA and qPCR analyses of orthotopic tumor tissues further showed that MAGI3 overexpression reduced CCL2 protein abundance in tumor tissues (Figure 3L) and was accompanied by decreased expression of the M2‐associated markers *Arg1*, *Tgfb1*, and *Il10*, together with increased expression of the M1‐associated markers *Tnf* and *Nos2* (Figure 3M). These changes were consistent with a shift of the macrophage phenotype toward a less immunosuppressive state. Consistent with these findings, *Magi3* overexpression markedly reduced tumor burden in the MC38 syngeneic tumor model (Figures 3N,O). It also decreased *Ccl2* transcript levels in tumor tissues (Figure 3P), accompanied by downregulation of *Arg1, Tgfb1*, and *Il10* and upregulation of *Tnf* and *Nos2* (Figure 3Q). Flow cytometric analysis further confirmed that MAGI3 overexpression significantly decreased the proportion of CD11b^+^ F4/80^+^ CD206^+^ M2‐like macrophages within tumors (Figure 3R). Conversely, *Magi3* knockdown in the CT26 syngeneic tumor model increased tumor‐associated CCL2 protein levels (Figure 3S), together with increased expression of *Arg1*, *Tgfb1*, and *Il10* and decreased expression of *Tnf* and *Nos2* (Figure 3T). Collectively, these in vivo findings indicate that MAGI3 restrains CCL2 production within tumors and limits M2‐like TAM recruitment and polarization.

To determine whether TAMs are required for tumor progression induced by tumor cell‐intrinsic MAGI3 deficiency, we performed macrophage depletion rescue experiments in *Magi3* knockout models. CT26 cells with CRISPR/Cas9‐mediated *Magi3* knockout were implanted into BALB/c mice to establish syngeneic tumor models, followed by systemic macrophage depletion using clodronate liposomes. Immunohistochemical analysis confirmed that clodronate treatment markedly reduced F4/80^+^ macrophage infiltration in tumor tissues (Figure 3U). Importantly, macrophage depletion abolished the tumor‐growth advantage conferred by MAGI3 loss in syngeneic tumor models established with *Magi3*‐knockout cells (Figures 3V,W). Collectively, these findings demonstrate that TAMs are essential contributors to tumor progression associated with MAGI3 deficiency.

Together, these results indicate that epithelial MAGI3 restrains the inflammation‐to‐cancer transition by limiting CCL2‐mediated recruitment and immunosuppressive reprogramming of TAMs.

### 3.3 MAGI3 Suppresses TAM Recruitment Through Inhibition of STAT3 Phosphorylation

To elucidate how MAGI3 regulates CCL2‐driven recruitment of M2‐like TAMs in CRC, we intersected the DEGs identified from tumors with high versus low MAGI3 expression in GSE72970 and GSE40967 datasets with gene sets associated with TAMs infiltration‐related pathways. This analysis yielded a total of 69 DEGs associated with TAM recruitment (Figure 4A), which were predominantly enriched in the JAK/STAT pathway (Figure 4B). The STAT family comprises STAT1, STAT2, STAT3, STAT4, STAT5A, STAT5B, and STAT6 and serves as a core group of transcription factors mediating cytokine signaling, thereby playing pivotal roles in inflammatory responses and tumorigenesis [35]. Given the enrichment of the JAK/STAT pathway, we next systematically evaluated the association between the activity of each STAT member and MAGI3 expression. Notably, among all STAT family members, only STAT3 activity showed a significant inverse correlation with MAGI3 (Figure S9A–F). Consistently, gene set enrichment analysis (GSEA) further confirmed that STAT3 signaling was significantly enriched in the MAGI3‐low patient group (Figures 4C–F). Moreover, PCA of STAT3 target genes clearly separated tumor from normal tissues, with MAGI3 expression inversely associated with STAT3 activity, as indicated by the first principal component (PCA1) representing STAT3 pathway activity (Figures 4G,H).

**FIGURE 4:**
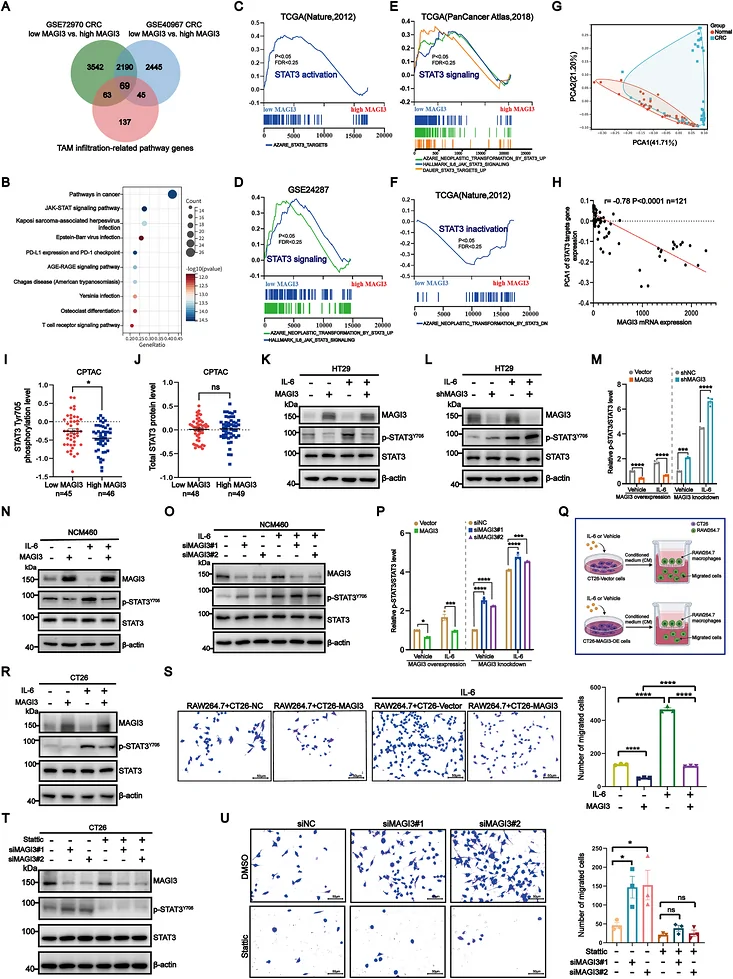
MAGI3 suppresses CCL2‐driven macrophage recruitment by inhibiting STAT3 Tyr705 phosphorylation. **A**. Identification of 69 common DEGs by intersecting genes differentially expressed between MAGI3‐low and MAGI3‐high tumors from the GSE72970 and GSE40967 datasets with TAM infiltration‐associated genes. DEGs were defined by |log_2_FC| > 0.2 and p < 0.05 in both datasets. **B**. GO analysis of biological processes associated with the 69 DEGs. **C‐F**. Enrichment plots of GSEA showed that the STAT3‐activated gene signature was enriched in the MAGI3‐low group, whereas the STAT3‐repressed gene signature was enriched in the MAGI3‐high group. **G**. Principal component analysis (PCA) of STAT3 target gene expression profiles in Normal and CRC samples. **H**. Pearson correlation between MAGI3 mRNA expression and PC1 of STAT3 target‐gene expression in CRC specimens (n = 121). **I**. STAT3 Tyr705 phosphorylation levels measured using CPTAC phosphoproteomic data in MAGI3‐low (n = 45) and MAGI3‐high (n = 46) CRC tumors (Mann‐Whitney U test). **J**. Total STAT3 protein abundance analyzed using CPTAC proteomic data in CRC tumors stratified into MAGI3‐low (n = 48) and MAGI3‐high (n = 49) groups according to MAGI3 protein abundance (Mann‐Whitney U test). **K‐L**. Western blot analysis of MAGI3, p‐STAT3^Y705^, and STAT3 protein levels in HT29 cells with MAGI3 overexpression **(K)** or shRNA‐mediated MAGI3 knockdown **(L)**, following treatment with ddH_2_O or IL‐6 (5 ng/mL, 30 min). β‐actin was used as a loading control. n = 3 independent biological replicates. **M**. Quantification of p‐STAT3^Y705^/STAT3 ratios in HT29 cells with overexpressing MAGI3 or subjected to MAGI3 knockdown, with or without IL‐6 stimulation (5 ng/mL, 30 min). Statistical significance was assessed using one‐way ANOVA followed by Tukey's multiple‐comparisons test. n = 3 independent biological replicates. **N‐O**. Western blot analysis of MAGI3, p‐STAT3^Y705^, and STAT3 protein levels in NCM460 cells transfected with MAGI3 overexpression plasmid **(N)** or two independent MAGI3 siRNAs **(O)** under ddH_2_O or IL‐6 (5 ng/ml, 30 min) treatment conditions. β‐actin was used as a loading control. n = 3 independent biological replicates. **P**. Quantification of p‐STAT3^Y705^/STAT3 ratios in NCM460 cells transfected with MAGI3 overexpression plasmid or two independent MAGI3 siRNAs, with or without IL‐6 stimulation (5 ng/mL, 30 min). Statistical significance was assessed using one‐way ANOVA followed by Tukey's multiple‐comparisons test. n = 3 independent biological replicates. **Q**. The co‐culture schematic shows the collection of conditioned medium (CM) from CT26‐Vector or CT26‐MAGI3 cells treated with ddH_2_O or IL‐6, followed by its use in RAW264.7 macrophage migration assays. **R**. Western blot analysis of p‐STAT3^Y705^, STAT3, and MAGI3 in CT26‐Vector and CT26‐MAGI3 cells treated with ddH_2_O or IL‐6 stimulation. n = 3 independent biological replicates. **S. Left**: After treatment with or without IL‐6 stimulation, the effect of conditioned medium (CM) from the CT26‐Vector/MAGI3 cells on regulatory macrophage recruitment was detected by performing a transwell migration assay. Scale bar, 50 µm. **Right**: Quantitative analysis of RAW264.7 cells cultured with IL‐6 treated with untreated Vector and MAGI3 cells. Migrated cells were quantified within 200 µm fields of view. Statistical significance was determined using one‐way ANOVA with Tukey's multiple comparisons test. n = 3 independent biological replicates. **T**. Western blot analysis of p‐STAT3^Y705^, STAT3, and MAGI3 in CT26‐siNC, CT26‐siMAGI3#1, and CT26‐siMAGI3#2 cells treated with DMSO or Stattic. n = 3 independent biological replicates. **U. Left**: Transwell migration assay assessing the effect of conditioned medium (CM) from Stattic‐treated or untreated CT26‐siNC, CT26‐siMAGI3#1, and CT26‐siMAGI3#2 cells on RAW264.7 macrophage migration. Representative images are shown. Scale bars: 50 µm. **Right**: Quantitative analysis of RAW264.7 cells cultured with Stattic‐treated and untreated CT26‐siNC, CT26‐siMAGI3#1, and CT26‐siMAGI3#2 cells. Migrated cells were quantified within 200 µm fields of view. Statistical significance was determined using one‐way ANOVA with Tukey's multiple comparisons test. n = 3 independent biological replicates. All quantitative data are presented as mean ± SEM. ^*^ *p* < 0.05, ^**^ *p* < 0.01, ^***^ *p* < 0.001, ^****^ *p < 0.0001; ns, not significant*.

To dissect the mechanism underlying MAGI3‐mediated regulation of STAT3, we first examined their correlation in CRC datasets and found no significant association at the transcriptional level (Figure S10A–G), suggesting a post‐transcriptional mechanism. Analysis of CPTAC phosphoproteomic data showed that STAT3 Tyr705 phosphorylation was lower in tumors with high MAGI3 abundance than in those with low MAGI3 abundance (Figure 4I). In contrast, analysis of CPTAC proteomic data showed no significant difference in total STAT3 protein abundance between the two groups (Figure 4J). Consistent with these findings, CHX chase assays showed that MAGI3 manipulation did not appreciably alter STAT3 degradation kinetics, indicating that the observed changes in STAT3 phosphorylation were unlikely to result from altered STAT3 protein stability (Figure S11A–B). To validate these observations experimentally, we examined STAT3 activation in the CRC cell line HT29 and the normal colonic epithelial cell line NCM460. In HT29 cells, MAGI3 overexpression reduced basal STAT3 Tyr705 phosphorylation, whereas MAGI3 knockdown increased it; these regulatory effects were further enhanced by moderate IL‐6 stimulation (Figures 4K–M). Similarly, in NCM460 cells, MAGI3 overexpression decreased basal STAT3 Tyr705 phosphorylation, whereas MAGI3 knockdown using two independent siRNAs increased it. IL‐6 stimulation further amplified these MAGI3‐dependent differences (Figures 4N–P). Together, these results indicate that MAGI3 modulates STAT3 activation, with its regulatory effects becoming more apparent upon IL‐6 stimulation.

Next, we co‐cultured RAW264.7 macrophages with conditioned medium from CT26 cells to explore whether MAGI3‐mediated regulation of TAM recruitment is mediated through STAT3 phosphorylation (Figure 4Q). Conditioned medium from *Magi3*‐overexpressing CT26 cells markedly reduced macrophage migration, with a more pronounced effect following IL‐6 stimulation (Figures 4R,S). Conversely, inhibition of STAT3 with Stattic reversed the increased macrophage recruitment caused by MAGI3 knockdown (Figures 4T,U), supporting the involvement of STAT3 signaling in MAGI3‐dependent regulation of macrophage migration.

To further determine whether CCL2 functions as a critical downstream effector of MAGI3–STAT3 signaling in vivo, we established CT26 syngeneic tumors using control or *Magi3*‐deficient cells and treated tumor‐bearing mice with either a CCL2‐neutralizing antibody or an isotype‐matched control IgG. *Magi3* deficiency markedly accelerated tumor growth under isotype‐matched IgG treatment, whereas CCL2 neutralization substantially attenuated the tumor growth advantage associated with *Magi3* deficiency (Figure S12A–C). Immunohistochemical analysis further showed that CCL2 blockade reduced CD206^+^ TAM accumulation induced by *Magi3* deficiency, whereas increased STAT3 Tyr705 phosphorylation in *Magi3*‐deficient tumors was maintained (Figure S12D). These findings support a functional role for CCL2 as a downstream effector of MAGI3–STAT3 signaling in promoting TAM accumulation and tumor progression.

Collectively, these findings indicate that MAGI3 modulates STAT3 activation and limits CCL2‐associated macrophage recruitment, thereby restraining tumor‐promoting macrophage accumulation and tumor progression.

### 3.4 MAGI3 Suppresses STAT3 Activation Through PDZ1‐Mediated Disruption of JAK2‐STAT3 Signaling

MAGI3 contains five PDZ domains, facilitating multi‐protein complex assembly [36]. In addition, STAT3 harbors a potential PDZ domain binding motif (PBM) at its C‐terminus (T‐S‐P‐M) [37, 38], suggesting a potential interaction with MAGI3.

To determine whether MAGI3 directly interacts with STAT3, we performed Co‐IP assays in the CRC cell lines HCT116 and HT29, as well as in the normal colonic epithelial cell line NCM460 (Figures 5A–C). The results demonstrated an interaction between MAGI3 and STAT3. Subsequently, an endogenous Co‐IP assay using lysates from normal mouse intestinal tissues further corroborated these findings (Figure 5D). Furthermore, immunofluorescence analysis revealed cytoplasmic co‐localization of MAGI3 and STAT3 (Figure 5E). Next, GST pull‐down assays further confirmed a direct interaction between the PDZ1 domain of MAGI3 and STAT3 (Figure 5F).

**FIGURE 5:**
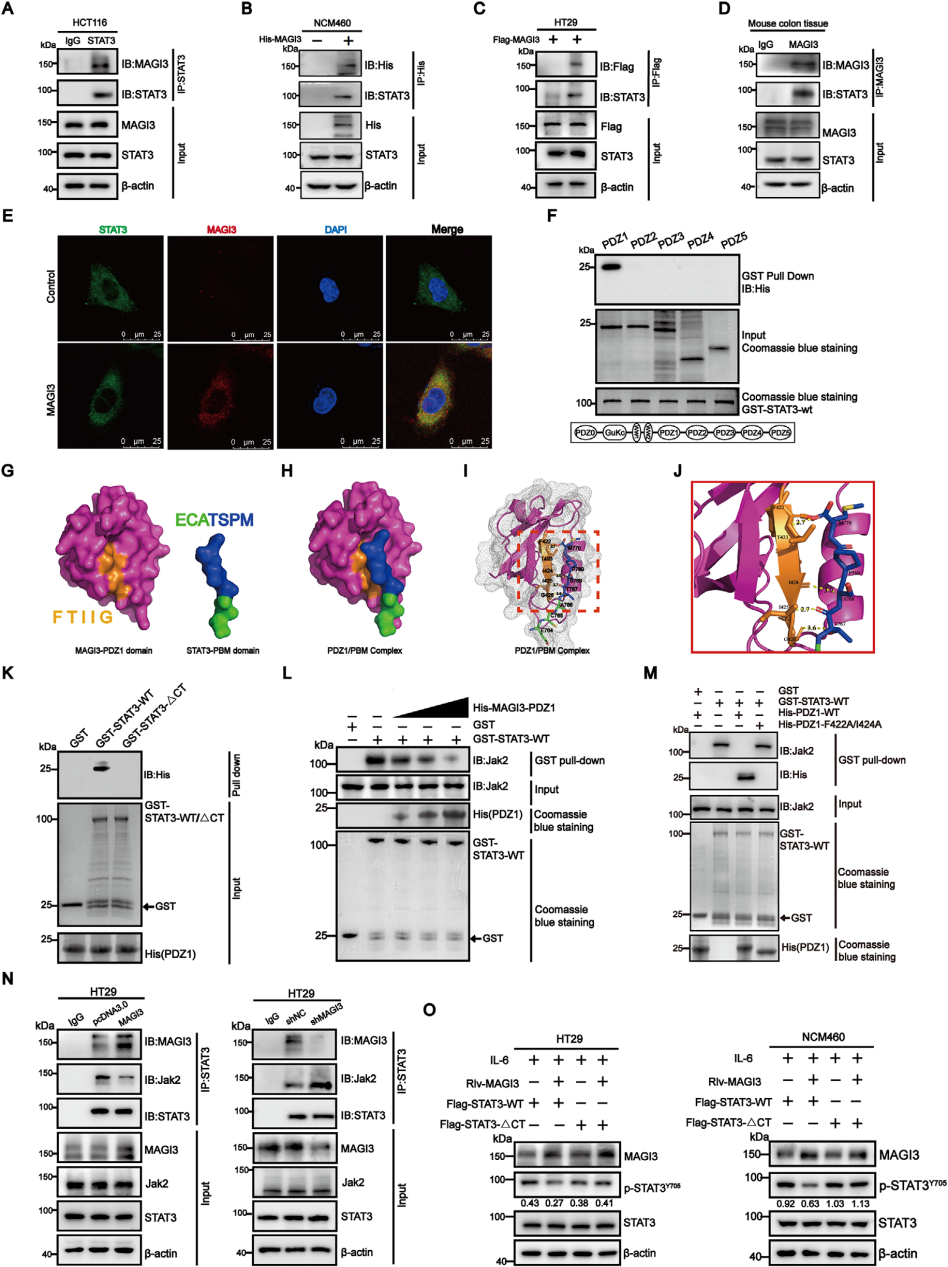
MAGI3 suppresses STAT3 activation by binding the STAT3 C‐terminal PBM via its PDZ1 domain and disrupting JAK2‐STAT3 signaling. **A**. Reciprocal Co‐IP was performed to detect the association between MAGI3 and STAT3 in HCT116 cells. n = 3 independent biological replicates. **B–C**. Co‐immunoprecipitation analysis of the interaction between exogenous MAGI3 and endogenous STAT3 in NCM460 **(B)** and HT29 **(C)** cells. Lysates from NCM460 cells transfected with an empty vector or a His‐MAGI3 expression plasmid were immunoprecipitated with an anti‐His antibody **(B)**. Lysates from HT29 cells expressing Flag‐MAGI3 were immunoprecipitated with control IgG or an anti‐Flag antibody **(C)**. Immunoprecipitates were immunoblotted for STAT3 and the corresponding tag (His or Flag). Input lysates were analyzed for tagged MAGI3, STAT3, and β‐actin. n = 3 independent biological replicates. **D**. Colonic tissue lysates from mice were immunoprecipitated with anti‐MAGI3 antibodies, and the interaction with STAT3 was analyzed by western blot. n = 3 independent biological replicates. **E**. NCM460 cells were transfected with control or Flag‐MAGI3 plasmids, and STAT3‐MAGI3 co‐localization was visualized by immunofluorescence. n = 3 independent biological replicates. **F**. Pull‐down analysis of the interaction between GST‐STAT3‐WT and His‐PDZ1‐5. The schematic representation of the domain structure of MAGI3 (bottom). n = 3 independent biological replicates. **G**. The predicted AlphaFold models of MAGI3‐PDZ1, where the carboxylate‐binding loop (Phe‐422, Thr‐423, Ile‐424, Ile‐425, and Gly‐426) is colored in orange, and the other residues are colored in magenta. The STAT3‐PBM is colored in blue (Thr‐767, Ser‐768, Pro‐769, Met‐770) and green (Glu‐764, Cys‐765, Ala‐766). **H**. The molecular simulation of the MAGI3‐PDZ1/STAT3‐PBM complex. Front view of the interaction between MAGI3‐PDZ1/STAT3‐PBM. **I‐J**. Stereoview of the detailed interface of the MAGI3‐PDZ1/STAT3‐PBM complex. Dotted lines represent chemical bonds. Right panels **(J)** show magnifications of the wireframe areas on the left **(I)**. The “FTIIG” motif of MAGI3‐PDZ1 and the “TSPM” motif of STAT3 are labeled. **K**. Pull‐down analysis of the interaction between GST‐STAT3‐WT, GST‐STAT3‐△CT, and His‐PDZ1. n = 3 independent biological replicates. **L**. Recombinant MAGI3‐PDZ1 protein disrupts the endogenous JAK2‐STAT3 interaction in a dose‐dependent manner. Endogenous JAK2 from HT29 cell lysates was pulled down using 10 µg of GST‐STAT3‐WT beads in the presence of increasing volumes of purified His‐MAGI3‐PDZ1 protein (0, 30, 35, 40 µg). JAK2 in the complexes was detected by western blotting, showing that the PDZ1 domain of MAGI3 competitively inhibits the STAT3‐JAK2 interaction. n = 3 independent biological replicates. **M**. GST pull‐down assays demonstrating the functional importance of PDZ1 residues F422 and I424 in STAT3 binding. GST or GST‐STAT3‐WT proteins were incubated with His‐PDZ1‐WT or His‐PDZ1‐F422A/I424A (F422A/I424A double mutant), followed by GST pull‐down and immunoblotting analysis of JAK2 and His‐tagged proteins. **N**. In HT29 cells, overexpressed MAGI3 decreased the interaction between STAT3 and its kinase JAK2 (left), whereas shMAGI3 increased the interaction between STAT3 and its kinase JAK2 (right). n = 3 independent biological replicates. **O**. Overexpression of MAGI3 transfected with Flag‐STAT3‐WT decreased phosphorylation of STAT3, but not of the Flag‐STAT3‐△CT transfected, both in HT29 (left) and NCM460 (right) cells after IL‐6 protein treated with 12 h. n = 3 independent biological replicates.

Consistently, AlphaFold‐based modeling indicated that the C‐terminal PBM of STAT3 docks into the hydrophobic pocket of MAGI3‐PDZ1, with predicted hydrogen bonds and hydrophobic contacts contributing to the interaction (Figures 5G–J, Table S1). Moreover, truncation of the STAT3 PBM abolished its interaction with MAGI3 (Figure 5K), confirming a direct interaction between the first PDZ domain of MAGI3 and the STAT3 PBM.

JAK kinases (JAK1/2/3, TYK2) phosphorylate STAT3 upon cytokine receptor engagement, with JAK2 being primary activators of IL‐6R/gp130‐mediated STAT3 signaling [39]. We further performed enrichment analysis of the JAK kinase pathway, which showed that MAGI3 expression was negatively correlated with JAK2‐regulated target genes (Figure S13A). In contrast, MAGI3 expression was positively correlated with JAK3‐regulated target genes and showed no correlation with those regulated by JAK1 or TYK2 (Figure S13B–D). Therefore, we hypothesized that MAGI3 binding interferes with JAK2‐STAT3 complex formation. To test this hypothesis, we next performed GST pull‐down and Co‐IP assays. In GST pull‐down assays, increasing concentrations of His‐MAGI3‐PDZ1 disrupted endogenous JAK2‐STAT3 binding in a dose‐dependent manner (Figure 5L). To further define the role of key amino acid residues within the MAGI3 PDZ1 domain in disrupting the JAK2‐STAT3 interaction, we generated a PDZ1 mutant carrying the F422A and I424A substitutions. GST pull‐down assays showed that wild‐type PDZ1 bound to STAT3 and markedly weakened the JAK2‐STAT3 interaction. In contrast, the PDZ1‐F422A/I424A double mutant largely lost its ability to bind STAT3 and failed to effectively disrupt formation of the JAK2‐STAT3 complex (Figure 5M). These findings indicate that F422 and I424 are critical residues required for STAT3 binding by the MAGI3 PDZ1 domain and for interference with JAK2–STAT3 binding.

Consistently, Co‐IP assays showed that overexpression of His‐MAGI3 in HT29 cells reduced the binding of STAT3 to JAK2, whereas MAGI3 knockdown enhanced this interaction (Figure 5N). Furthermore, experiments in NCM460 and HT29 cells confirmed that MAGI3 overexpression selectively inhibited phosphorylation of STAT3‐wt but not STAT3‐Δct (lacking the C‐terminal PBM; Figure 5O).

To further clarify how MAGI3 inhibits JAK‐mediated STAT3 phosphorylation, we performed protein structure modeling of this interaction. Upon JAK2‐mediated phosphorylation, STAT3 homodimerizes through reciprocal SH2‐pTyr interactions and translocates to the nucleus to regulate transcription (Figure S14A–C). Structural modeling suggested that MAGI3‐PDZ1 binding at the STAT3 C‐terminus may interfere with conformational accessibility of the SH2 domain required for STAT3 activation (Figure S14D–F), likely preventing STAT3 engagement with IL‐6R/gp130 and thereby blocking JAK2‐mediated phosphorylation (Figure S14G,H).

Overall, our results identify MAGI3 as an endogenous negative regulator of STAT3 activation that acts upstream of STAT3 phosphorylation, consistent with its role as an intestinal epithelial gatekeeper during inflammation‐associated carcinogenesis. By binding STAT3 via its PDZ1 domain, MAGI3 disrupts JAK2‐STAT3 interactions and impairs STAT3 phosphorylation, thereby suppressing STAT3‐dependent CCL2 production and TAM recruitment within the TME.

### 3.5 Tumor Cell‐Intrinsic MAGI3 Restrains STAT3‐CCL2 Signaling to Limit M2‐Like TAM Recruitment and Reshape TAM States Within the TME

To further corroborate the presence of a MAGI3‐mediated STAT3‐CCL2‐TAM axis in vivo, we employed the AOM/DSS‐induced inflammation‐associated CRC model with *Magi3^−/−^* mice. The results showed that *Magi3^−/−^* mice exhibited elevated p‐STAT3^Y705^, increased CCL2 expression, enhanced infiltration of CD206^+^/CD163^+^ TAMs, and a higher proliferative index (Ki67^+^) compared with controls (Figure S15A,B). Furthermore, Western blot analysis of tumor tissues confirmed higher p‐STAT3^Y705^ levels in tumors from *Magi3^−^/^−^* mice (Figure S15C). These findings indicate that MAGI3 deficiency promotes a STAT3‐hyperactive, M2‐like TAM‐enriched microenvironment that facilitates inflammation‐associated CRC development.

To investigate how MAGI3 regulates the tumor immune microenvironment within the native tumor niche, we established an orthotopic cecal implantation model using CT26‐vector or *Magi3*‐overexpressing cells and performed scRNA‐seq analysis of tumor tissues. Unsupervised clustering identified the major cellular compartments within the tumor ecosystem, including malignant cells, fibroblasts, endothelial cells, myeloid cells, and lymphoid cells (Figure 6A,B). The identity of these major cell populations was further validated based on the expression of canonical marker genes (Figure 6C). Across all cell populations, *Magi3* expression was preferentially enriched in malignant epithelial cells (Figure 6D) and was markedly increased in the *Magi3*‐overexpression group (Figure 6E), confirming the successful establishment of the overexpression model. Further analysis showed that *Magi3* overexpression significantly reduced STAT3 pathway activity scores (Figure 6F) and *Ccl2* expression (Figure 6G) in tumor cells, suggesting that MAGI3 may reduce tumor cell‐derived CCL2 production by inhibiting STAT3 signaling.

**FIGURE 6:**
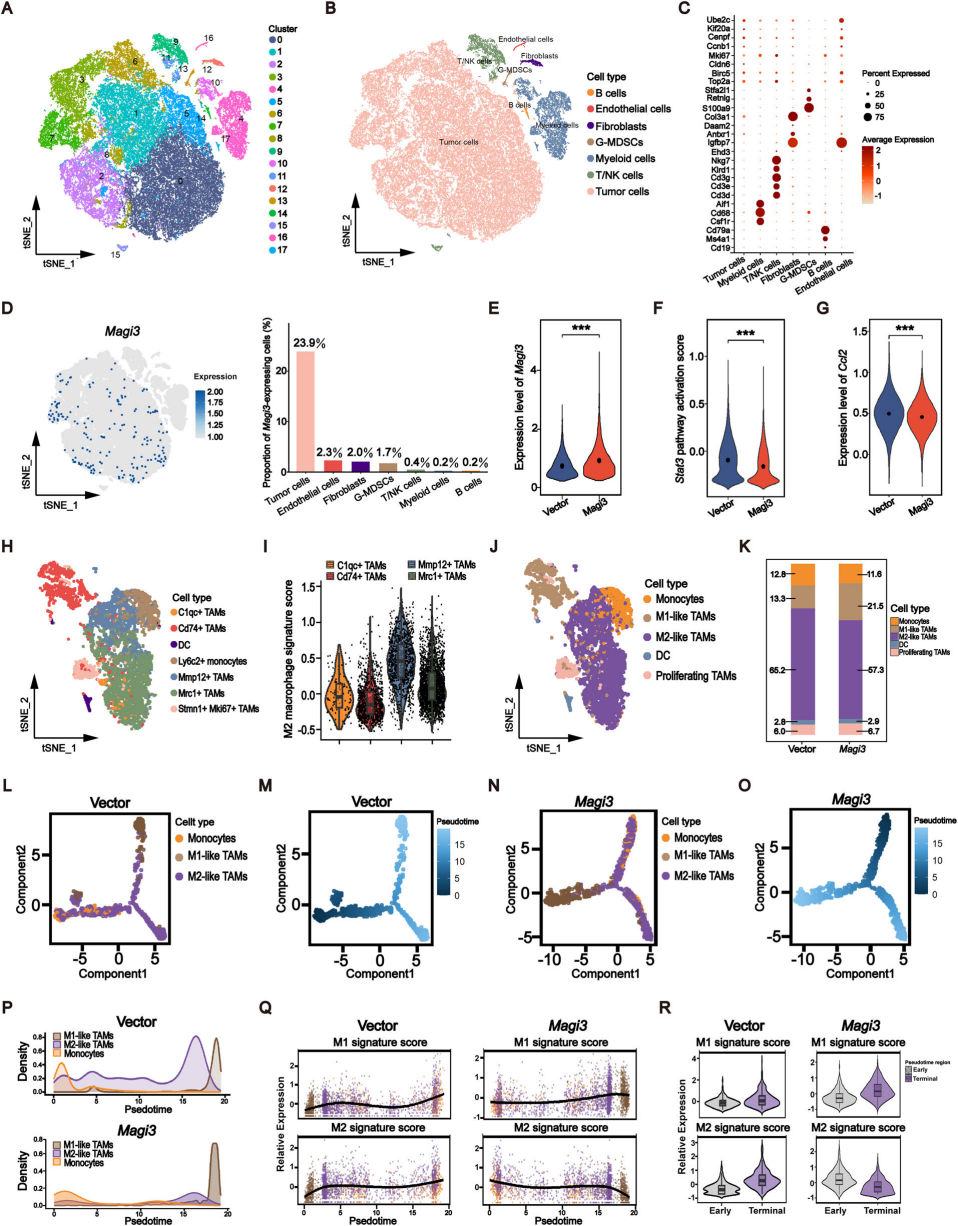
scRNA‐seq analysis of orthotopic tumors reveals that tumor cell‐intrinsic MAGI3 restrains STAT3–CCL2 signaling, limits M2‐like TAM recruitment, and reshapes macrophage states. **A‐C**. Single‐cell RNA sequencing analysis of mouse orthotopic tumors (n = 3 per group). t‐SNE visualization showing distinct cellular clusters identified from tumor samples (A). Annotation of major cell populations, including tumor cells, endothelial cells, fibroblasts, myeloid cells, T/NK cells, B cells, and G‐MDSCs (B). Dot plot showing the expression of representative marker genes used for cell‐type identification (C). **D**. Distribution and expression pattern of *Magi3* across different cell populations. **Left**: t‐SNE feature plot showing *Magi3* expression; **Right**: violin plot comparing *Magi3* expression levels among indicated cell types. **E‐G**. Characterization of MAGI3‐associated tumor cell phenotypes. *Magi3* expression levels in tumor cells from vector‐ and *Magi3*‐overexpressing tumors **(E)**. Activation score of the STAT3 signaling pathway in tumor cells between the indicated groups **(F)**. *Ccl2* expression levels in tumor cells **(G)**. Statistical significance in E‐G was assessed using a two‐tailed unpaired Student's t‐test. **H‐K**. Single‐cell analysis of TAM heterogeneity. t‐SNE visualization of TAM subpopulations, including C1qc^+^ TAMs, Cd74^+^ TAMs, Mmp12^+^ TAMs, Mrc1^+^ TAMs, and proliferating TAMs **(H)**. M2 macrophage signature scores across TAM subpopulations **(I)**. t‐SNE visualization of macrophage states classified into monocytes, M1‐like TAMs, M2‐like TAMs, dendritic cells, and proliferating TAMs **(J)**. Relative proportions of macrophage subsets in vector‐ and *Magi3*‐overexpressing tumors **(K)**. **L‐O**. Pseudotime trajectory analysis of TAM differentiation states. Distribution of macrophage subsets along pseudotime trajectories in vector‐ and *Magi3*‐overexpressing tumors **(L, N)**. Pseudotime progression of TAM states in the indicated groups **(M, O)**. **P**. Density distribution of pseudotime trajectories showing altered macrophage state transitions following *Magi3* overexpression. **Q**. Dynamic changes of M1‐ and M2‐like macrophage signature scores along pseudotime trajectories in vector‐ and *Magi3*‐overexpressing tumors. **R**. Violin plots showing M1 and M2 macrophage signature scores at early and terminal pseudotime states in vector‐ and *Magi3*‐overexpressing tumors. All quantitative data are presented as mean ± SEM. ^***^ *p* < 0.001, ^****^ *p < 0.0001*.

We next characterized the myeloid compartment to determine whether MAGI3‐mediated alterations in tumor cell signaling influence macrophage composition and functional states. Unsupervised clustering identified distinct myeloid cell subsets, including C1q^+^ TAMs, Cd74^+^ TAMs, dendritic cells (DCs), Ly6c2^+^ monocytes, Mmp12^+^ TAMs, Mrc1^+^ TAMs, and Stmn1^+^Mki67^+^ TAMs (Figure 6H). Based on M2 macrophage signature scores across TAM subpopulations (Figure 6I) and canonical M1/M2 macrophage‐associated markers, macrophage populations were further reclassified to distinguish M1‐like TAMs from M2‐like TAMs (Figure 6J). Cell proportion analysis demonstrated that *Magi3* overexpression reduced the proportion of M2‐like TAMs while increasing that of M1‐like TAMs within the tumor microenvironment (Figure 6K).

To further elucidate the impact of MAGI3 on macrophage differentiation trajectories, we performed Monocle2‐based pseudotime trajectory analysis of monocytes and macrophages. The trajectory analysis suggested that monocytes progressively differentiated toward M1‐like and M2‐like macrophage states. Compared with vector‐control tumors (Figure 6L,M), *Magi3*‐overexpressing tumors exhibited reduced accumulation of cells at terminal M2‐like differentiation states, accompanied by increased enrichment of M1‐like macrophages along the differentiation trajectory (Figure 6N,O). Pseudotime density distribution analysis further confirmed that *Magi3* overexpression induced a distinct shift in macrophage differentiation patterns (Figure 6P). Consistently, along pseudotime progression, *Magi3*‐overexpressing tumors displayed decreased M2‐associated signatures and enhanced M1‐associated signatures compared with vector controls, indicating that MAGI3 promotes macrophage polarization toward an M1‐like inflammatory phenotype (Figure 6Q,R).

Together, these AOM/DSS and orthotopic scRNA‐seq findings support the MAGI3‐STAT3‐CCL2‐TAM axis as an important regulator of inflammation‐associated colorectal tumorigenesis and indicate that tumor cell‐intrinsic MAGI3 limits M2‐like TAM recruitment and reprograms macrophage states, thereby shaping the immune architecture of the TME.

### 3.6 The MAGI3‐STAT3 Axis Restrains CRC Progression by Limiting M2‐Like TAM Recruitment

To determine the clinical significance of the MAGI3‐STAT3‐CCL2‐TAM axis, we evaluated whether MAGI3 loss in human CRC specimens was associated with STAT3 hyperactivation, increased CD206^+^ TAM infiltration, and adverse patient outcomes. IHC analysis of CRC samples (Table S2) simultaneously assessed MAGI3 protein levels alongside p‐STAT3^Y705^ activation and CD206^+^ TAMs. Low MAGI3 expression was associated with higher p‐STAT3^Y705^ levels and increased CD206^+^ TAM infiltration (Figures 7A–C). Concordantly, patients with low MAGI3 expression exhibited a higher recurrence rate (Figure 7D) and a shorter relapse‐free survival (Figure 7E). Nomogram analysis also indicated that low MAGI3 and high CCL2 expression corresponded to poorer predicted overall survival (OS) and progression‐free survival (PFS) (Figure S16A,B). Univariate Cox regression (Table S3) and multivariate Cox regression (Table S4) further confirmed MAGI3 as an independent prognostic factor for CRC outcomes.

**FIGURE 7:**
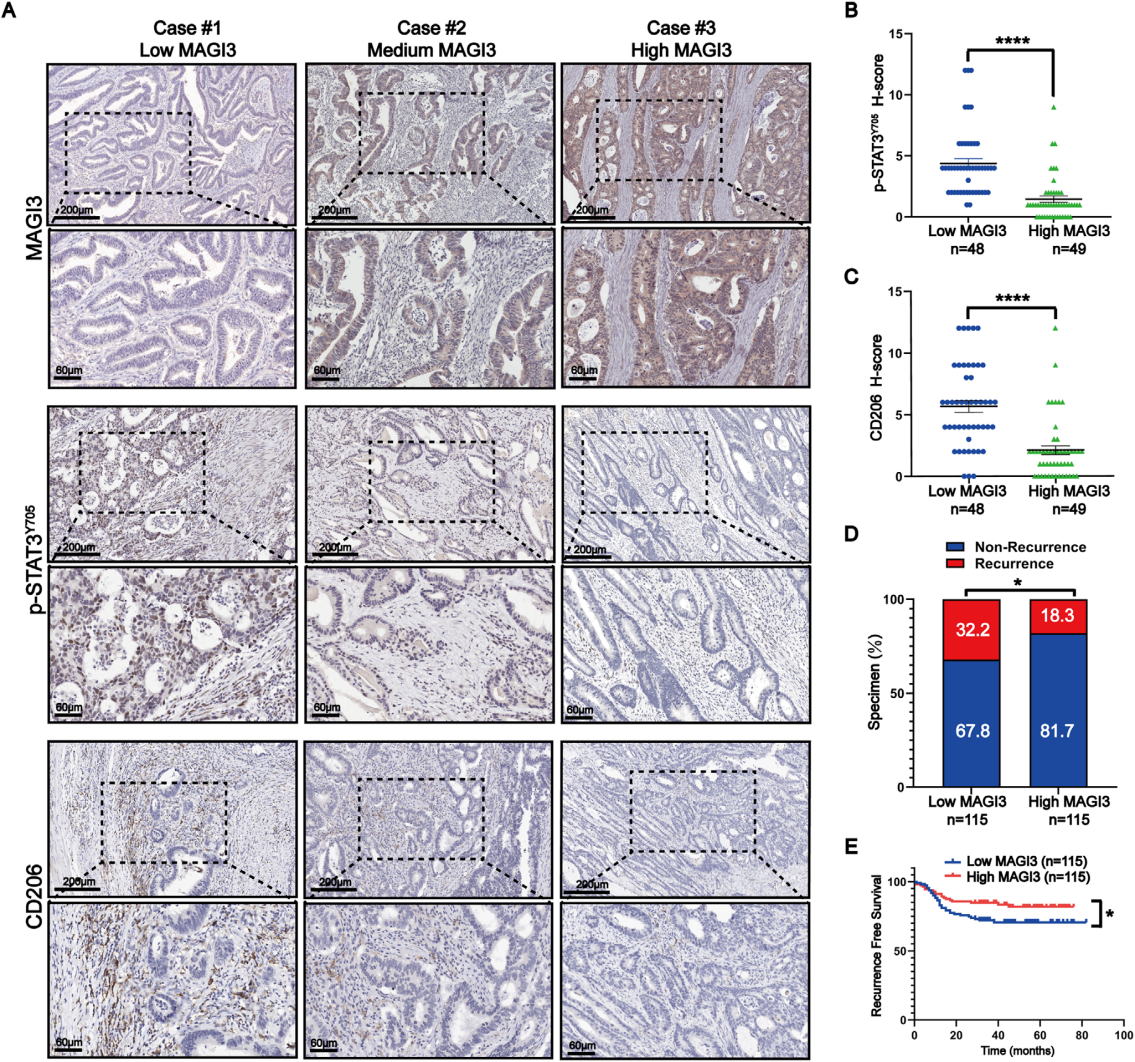
Low MAGI3 expression is associated with STAT3 hyperactivation, M2‐like TAM enrichment, and poor prognosis in CRC. **A‐C**. Immunohistochemistry (IHC) was performed on human CRC specimens. Representative images of MAGI3, p‐STAT3 ^Y705^, and CD206 staining across different MAGI3 expression levels **(A)**, quantification of H‐scores for p‐STAT3 ^Y705^ **(B)**, and quantification of H‐scores for CD206 **(C)**. Statistical analysis was performed using the Mann‐Whitney U test. n = 48 patients/Low MAGI3 group, n = 49 patients/High MAGI3 group. These groups represent subsets of the 230 patients, with the grouping based on the median MAGI3 IHC staining score, categorizing patients into Low and High expression groups for further analysis. **D‐E**. Clinical outcomes: recurrence rate **(D**, Chi‐square test) and recurrence‐free survival **(E**, Kaplan‐Meier curves, log‐rank test) stratified by MAGI3 expression. n = 115 patients/group. All quantitative data are presented as mean ± SEM. ^*^ *p* < 0.05, ^****^ *p < 0.0001*.

Together, these results highlight a clinically relevant subset of CRC patients characterized by low MAGI3 expression, STAT3 hyperactivation, and enrichment of M2‐like TAMs, which is closely associated with poor prognosis and chemoresistance.

### 3.7 MAGI3 Deficiency Confers Enhanced Sensitivity to STAT3 Inhibition

Given that STAT3 hyperactivation and TAM infiltration contribute to resistance to chemotherapy and immunotherapy [40, 41], we next assessed the impact of MAGI3 expression levels on responses to STAT3‐targeted therapy to determine whether MAGI3 status influences sensitivity to STAT3‐targeted therapy. To this end, we established subcutaneous syngeneic tumor models using control or CRISPR/Cas9‐mediated *Magi3*‐knockout CT26 cells, followed by daily treatment with vehicle or the STAT3 inhibitor Stattic (Figure 8A). Under vehicle treatment, *Magi3* loss markedly increased tumor weight (Figures 8B,C) and accelerated tumor growth (Figure 8D). Stattic treatment substantially suppressed tumor growth in both groups and abolished the growth advantage conferred by *Magi3* loss, with no significant difference in tumor weight or volume between control and *Magi3*‐knockout tumors after Stattic treatment. Notably, tumor growth inhibition (TGI) was significantly greater in *Magi3*‐knockout tumors than in control tumors (Figure 8E), suggesting that *Magi3* loss confers enhanced sensitivity to STAT3 inhibition.

**FIGURE 8:**
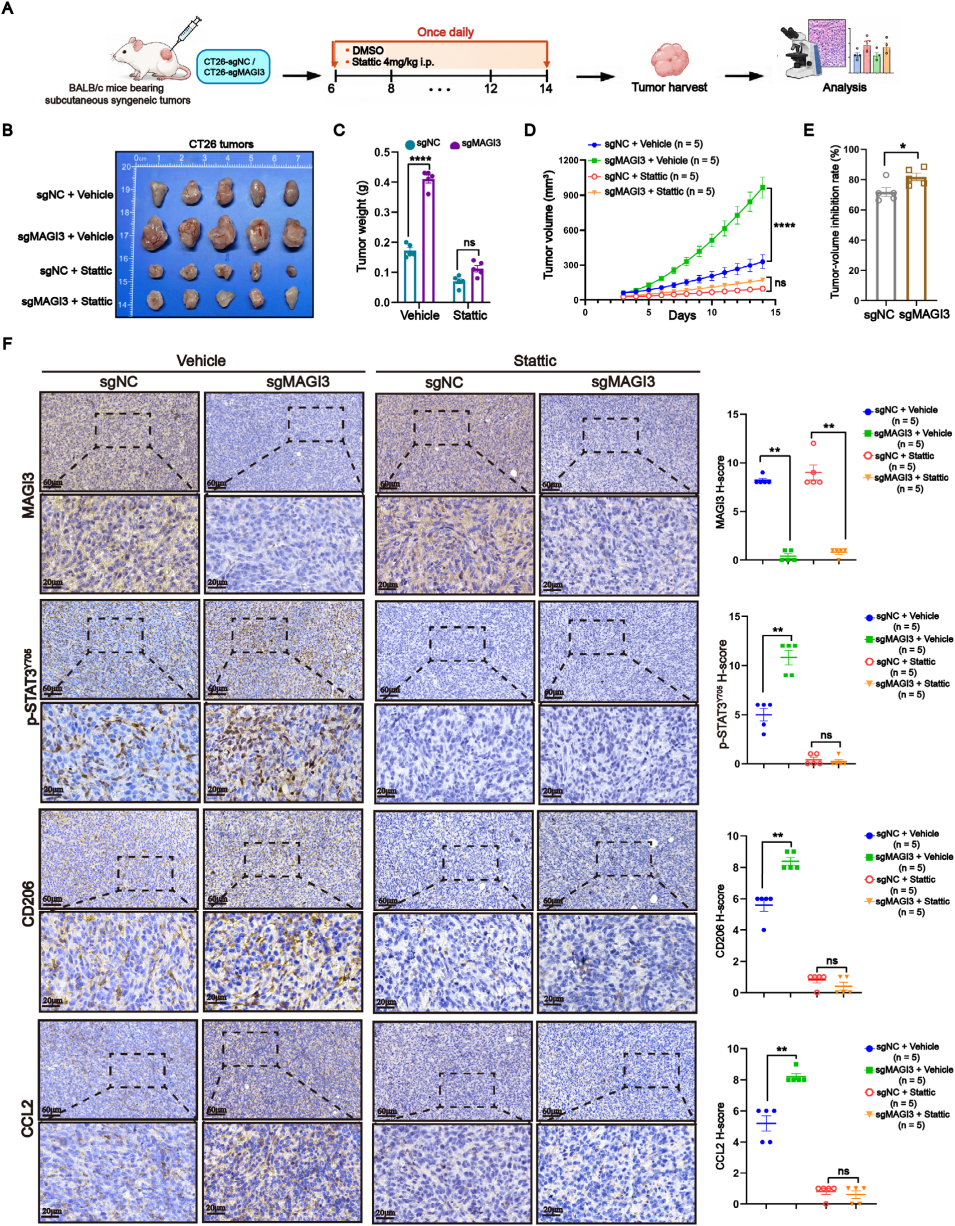
MAGI3 deficiency confers enhanced sensitivity to pharmacologic STAT3 inhibition. **A‐E**. Therapeutic evaluation of STAT3 inhibition in *Magi3*‐deficient CT26 syngeneic tumors. BALB/c mice bearing subcutaneous CT26 tumors were treated with vehicle or the STAT3 inhibitor Stattic (4 mg/kg, intraperitoneally, once daily). **A**. Schematic of the treatment schedule. **B**. Representative tumor images from each group are shown. **C**. Tumor weights were quantified after treatment (two‐tailed unpaired Student's t‐test; n = 5 mice/group). **D**. Tumor growth curves of mice bearing sgNC or sgMAGI3 CT26 tumors treated with vehicle or Stattic (two‐way ANOVA with Šidák's multiple comparisons test; n = 5 mice/group). **E**. Tumor growth inhibition rates following Stattic treatment in sgNC and sgMAGI3 tumors (two‐tailed unpaired Student's t‐test; n = 5 mice/group). **F**. Subcutaneous tumor samples from animals treated with Stattic were quantified for H‐scores of p‐STAT3^Y705^, CCL2, MAGI3, and CD206. n = 5 mice/group. Statistical significance was determined using the Mann‐Whitney U test. All quantitative data are presented as mean ± SEM. ^*^ *p* < 0.05, ^**^ *p* < 0.01, ^***^ *p* < 0.001, ^****^ *p < 0.0001; ns, not significant*.

Consistently, immunohistochemical analysis revealed that *Magi3*‐deficient tumors exhibited higher levels of p‐STAT3^Y705^ levels, increased CCL2 expression, and enhanced CD206^+^ M2‐like TAM infiltration compared with control tumors. Stattic treatment markedly reduced p‐STAT3^Y705^ levels, CCL2 expression, and M2‐like TAM accumulation in *Magi3*‐deficient tumors, largely eliminating the differences between *Magi3*‐deficient and control groups (Figure 8F).

Taken together, these results demonstrate that MAGI3 deficiency promotes a STAT3‐dependent immunosuppressive tumor state and enhances tumor vulnerability to STAT3 inhibition, suggesting that MAGI3 may serve as a potential biomarker for stratifying CRC patients likely to benefit from STAT3‐targeted therapies.

## 4 Discussion

Chronic, cytokine‐driven inflammation is a well‐established hallmark contributing to colorectal tumorigenesis [42], yet how intestinal epithelial cells intrinsically determine whether inflammatory signals remain homeostatic or become tumor‑promoting remains poorly understood. In this study, we identify MAGI3 as a previously unrecognized intestinal epithelial gatekeeper that restrains inflammation‑associated CRC progression.

In support of this, analyses of multiple datasets, including human CRC cohorts, ulcerative colitis samples, and single‐cell transcriptomic profiles, revealed a stepwise downregulation of epithelial MAGI3 along the continuum from normal intestine to inflammatory bowel disease and ultimately to CRC. This progressive decrease in MAGI3 expression correlated with disease severity and was closely associated with poor prognosis in CRC. In an AOM/DSS‐induced model of inflammation‐associated CRC, *Magi3* knockout mice exhibited a marked increase in M2‐like macrophage infiltration within the tumor microenvironment, thereby facilitating the inflammation‐to‐cancer transition. Moreover, both in vivo and in vitro experiments showed that inflammatory cytokines can suppress MAGI3 expression. Collectively, these findings suggest that inflammation‐driven downregulation of epithelial MAGI3 represents an early molecular alteration associated with the transition from inflammatory homeostasis to tumor‐promoting inflammation, whereas sustained or further loss of MAGI3 promotes inflammation‐associated tumorigenesis and reinforces a self‐perpetuating vicious cycle. MAGI3, as a scaffolding protein, has been implicated in multiple malignancies. Previous studies have shown that MAGI3 exerts tumor‐suppressive effects by binding to PTEN and β‐catenin, thereby inhibiting glioma progression [43, 44]. In renal cell carcinoma, MAGI3 suppresses the proliferation of clear cell renal cell carcinoma (ccRCC) by interacting with MAS and inhibiting the MAS/ERK signaling axis [45]. In cervical cancer, MAGI3 also exerts anti‐tumor effects through the Wnt/β‐catenin pathway [46]. Collectively, these studies indicate that MAGI3 functions as a tumor suppressor in multiple cancers. However, our study identifies MAGI3 as a previously unrecognized epithelial gatekeeper during the inflammation‐to‐cancer transition in CRC.

Mechanistically, MAGI3 functions as a non‐canonical negative regulator of the IL‐6/JAK2/STAT3 pathway. Unlike previously described regulators such as PDLIM2 [47], which promotes STAT3 ubiquitination and proteasomal degradation, MAGI3 interacts with the C‐terminal region of STAT3 through its PDZ1 domain, thereby reducing JAK2‐mediated STAT3 phosphorylation and attenuating STAT3 activation (Figures 5G–J, Figure S14A–H). This spatial interference limits JAK2‐mediated phosphorylation of STAT3 at Tyr705, consequently suppressing downstream transcriptional programs, including CCL2 transcription. This mechanism is distinct from that of Pellino1, an E3 ubiquitin ligase that promotes and sustains pathogenic STAT3 signaling through K63‐linked ubiquitination of STAT3 [48], and from the circFAT1‐mediated mode of regulation, in which circFAT1 enhances STAT3 activity by preventing SHP1‐dependent dephosphorylation [49]. These comparisons further underscore MAGI3 as a distinct structural regulator of STAT3 signaling that restrains cytokine‐driven oncogenic signaling upstream at its source. Overall, our study expands the current understanding of the molecular mechanisms governing STAT3 phosphorylation.

Our previous work demonstrated that MAGI3 promotes c‐Myc degradation in CRC and has prognostic value in patients with CRC [50]. Building upon these findings, our present study demonstrates that MAGI3 deficiency also regulates the tumor immune microenvironment through the STAT3–CCL2 axis. These mechanisms are not mutually exclusive and may instead reflect the context‐dependent functions of MAGI3 within distinct tumor ecosystems. To further elucidate the functional heterogeneity of MAGI3, we performed CMS‐based stratification analysis using the TCGA CRC cohort. These analyses revealed subtype‐dependent patterns of signaling alterations associated with MAGI3 expression (Figure S17A–C). In CMS2 tumors, low MAGI3 expression was primarily associated with activation of MYC target gene programs, whereas IL6/JAK/STAT3 signaling was not significantly enhanced, suggesting that MAGI3 may predominantly regulate tumor cell‐intrinsic proliferative programs in this subtype (Figure S17D). In contrast, in the immune‐inflamed CMS1 subtype and the stroma‐enriched CMS4 subtype, low MAGI3 expression was more closely associated with activation of IL6/JAK/STAT3 signaling, suggesting that MAGI3 may contribute to the regulation of inflammation‐associated tumor ecosystems (Figure S17E).

Further stratification of the CPTAC cohort based on tumor inflammatory status revealed that the biological associations of MAGI3 loss are strongly influenced by the inflammatory context of CRC (Figure S18A). In high‐inflammation CRC tumors, low MAGI3 expression was associated with enhanced IL6/JAK/STAT3 signaling activity, but not with significant activation of the c‐MYC pathway, and was accompanied by enrichment of M2 macrophage‐related programs and immunosuppressive features (Figure S18B,D). Conversely, in low‐inflammation CRC tumors, low MAGI3 expression was associated with increased c‐MYC pathway activity but not STAT3 pathway activation, and was more closely linked to tumor cell‐intrinsic programs, including apoptosis and epithelial‐mesenchymal transition (EMT) (Figure S18C,E). Collectively, these findings suggest that MAGI3 is unlikely to exert its tumor‐suppressive effects through a single molecular pathway but instead functions as a context‐dependent regulator coordinating tumor cell‐intrinsic oncogenic signaling and immune microenvironmental remodeling. The previously identified MAGI3–c‐Myc axis and the MAGI3–STAT3–CCL2–TAM axis uncovered in this study together constitute a multilayered regulatory network through which MAGI3 restrains CRC progression. Thus, the biological functions of MAGI3 may be shaped by the molecular characteristics and microenvironmental states of individual tumors.

In addition, given the pronounced molecular heterogeneity of CRC, patient stratification requires careful consideration. Although our findings were consistent across multiple datasets and experimental systems, inter‐patient variability may still influence biomarker‐based stratification. CRC comprises distinct molecular subtypes defined by microsatellite instability (MSI), chromosomal instability (CIN), and key driver alterations such as *KRAS*, *TP53*, and *BRAF* ^V600E^ mutations, any of which could potentially confound predictions of therapeutic response. To address this, we systematically evaluated whether MAGI3 expression is influenced by MSI, CIN, or these oncogenic alterations across several independent cohorts (TCGA, GSE13294, GSE39582, and GSE39084) spanning broad genomic backgrounds (Figure S19A–K). Notably, MAGI3 expression was not significantly associated with these molecular subtypes and remained relatively stable across genomic subgroups, suggesting that its regulation is largely independent of these canonical genomic contexts.

Clinically, our previous study showed that MAGI3 expression can stratify CRC patients according to their response to adjuvant chemotherapy [50]. Patients with high MAGI3 expression had a low recurrence risk and did not require additional adjuvant chemotherapy, thereby avoiding overtreatment, whereas those with intermediate MAGI3 expression exhibited enhanced responsiveness to fluoropyrimidine‐based regimens (Figure S20A). However, therapeutic strategies for patients with low MAGI3 expression remain unclear. Within this subgroup, tumors with high inflammatory scores displayed pronounced STAT3 hyperactivation that was independent of c‐Myc signaling (Figure S20B–D) and were associated with a markedly higher rate of chemoresistance (65.8% vs. 38.5%; Figure S20E). Validation in an independent CRC cohort further confirmed that patients with high inflammation scores are more resistant to chemotherapy (Figure S20F) and experience significantly shortened relapse‐free survival after adjuvant therapy (Figure S20G). Moreover, our findings suggest that MAGI3 IHC in CRC specimens could serve as a practical biomarker for patient stratification. Multivariable analyses further confirmed MAGI3 as an independent prognostic factor and a potential predictor for chemotherapy response (Table S5). Taken together, our findings identify low MAGI3 expression and heightened inflammation in CRC as a clinically relevant subgroup, characterized by elevated STAT3 phosphorylation and an M2‐like TAM‐enriched TME, supporting further clinical investigation of STAT3 inhibitors in this molecularly defined subgroup.

Therapeutically, the MAGI3‐STAT3‐CCL2‐TAM axis defined in our study provides a mechanistic basis for selecting STAT3 inhibitors for patients with MAGI3‐low tumors. Prior TAM‐targeted strategies have largely relied on neutralizing antibodies targeting the CSF1/CSF1R pathway or CCL2, such as AMG 820 and carlumab. However, targeting the CSF1/CSF1R pathway can induce compensatory expansion of immunosuppressive cell populations, including Foxp3^+^ Treg cells [51]. Clinically, AMG 820, an anti‐CSF1R antibody, showed limited efficacy in combination with pembrolizumab [52]. Similarly, carlumab showed limited clinical activity in phase I/II trials, largely reflecting its transient suppression of free CCL2 and difficulty achieving sustained blockade [53, 54, 55]. In this context, stratifying patients by MAGI3 status and epithelial STAT3 activation status may help identify tumors most likely to respond to STAT3‐directed interventions targeting CCL2 production and TAM‐mediated immunosuppressive remodeling, providing a rationale for mechanism‐guided therapeutic development. Moreover, the experimentally defined MAGI3‐STAT3 interface provides a blueprint for developing first‐in‐class PDZ1‐based mimetics or synthetic stabilizers that may pharmacologically restore this intestinal epithelial regulatory function and suppress inflammation‐to‐cancer transition at its source.

Several limitations of this study warrant further consideration. Although we validated the MAGI3‐STAT3‐CCL2‐TAM axis across multiple dimensions, including clinical samples, in vivo models, and scRNA‐seq datasets, additional clinical cohorts are still needed to validate MAGI3 as a predictive biomarker for response to STAT3‐targeted therapy. Moreover, although Stattic demonstrated antitumor activity in preclinical models, its safety profile and potential toxicities require further evaluation.

Taken together, our study identifies MAGI3 as an intestinal epithelial gatekeeper that is persistently downregulated during the progression from normal colonic mucosa to inflamed tissue and ultimately to cancer. MAGI3 deficiency activates the STAT3‐CCL2 signaling, thereby promoting TAM‐mediated immunosuppression and inflammation‐associated colorectal tumor progression. These findings reveal an intestinal epithelial‐intrinsic immunoregulatory mechanism and provide a mechanistic and translational framework for targeting inflammation‐driven immune evasion in CRC.

## Author Contributions

**Yumeng Yang**: Conceptualization, Methodology, Investigation, Formal analysis, Data curation, Visualization, Writing – original draft. **Zhigang Bai**: Methodology, Software, Formal analysis, Validation, Visualization, Writing – original draft, Writing – review & editing. **Haibo Wang**: Investigation, Validation, Data curation, Visualization. **Yu Guo**: Investigation, Resources, Validation. **Yan Zhang**: Investigation, Data curation, Formal analysis. **Meihan Hu**: Investigation, Validation, Visualization. **Shuaikang Fan**: Formal analysis, Data curation, Visualization. **Ran Song**: Investigation, Resources, Data curation. **Tongmei Zhang**: Resources, Investigation, Validation. **Mengqi Deng**: Formal analysis, Software, Data curation, Visualization. **Xiaomei Yang**: Conceptualization, Supervision, Project administration, Funding acquisition, Writing – review & editing. **Zhongtao Zhang**: Conceptualization, Supervision, Project administration, Resources, Funding acquisition, Writing – review & editing. **Junqi He**: Conceptualization, Supervision, Project administration, Funding acquisition, Writing – review & editing.

## Funding

This work was supported by the Beijing Natural Science Foundation (No. L2610014 and 7222006), the National Natural Science Foundation of the People's Republic of China (No. 82673568 and 82273965).

## Materials Availability

All unique/stable reagents generated in this study will be made available on request, but we may require payment and/or a completed Materials Transfer Agreement if there is potential for commercial application.

## Compliance with Ethical Requirements

The study of human CRC samples was approved by the Ethics Committee of Beijing Friendship Hospital, Capital Medical University (approval number: 2018‐P2‐045‐01), and all patients provided informed consent. All animal procedures were conducted in accordance with the guidelines of the National Institutes of Health Guide for the Care and Use of Laboratory Animals, and the study was approved by the Capital Medical University Animal Use and Care Committee (approval numbers: AEEI‐2020‐133 and AEEI‐2025‐112). Animal experiments with subcutaneous syngeneic tumors were terminated when tumors did not exceed 1000 mm^3^. Consent for publication: All subjects provided written informed consent.

## Conflicts of Interest

The authors declare that they have no known competing financial interests or personal relationships that could have appeared to influence the work reported in this paper.

## Supporting information




**Supporting File 1**: advs78577‐sup‐0001‐Table1.docx.

**Supporting File 2**: advs78577‐sup‐0002‐Table2.docx.

**Supporting File 3**: advs78577‐sup‐0003‐Table1.docx.

**Supporting File 4**: advs78577‐sup‐0004‐Table2.docx.

**Supporting File 5**: advs78577‐sup‐0005‐Table3.docx.

**Supporting File 6**: advs78577‐sup‐0006‐Table4.docx.

**Supporting File 7**: advs78577‐sup‐0007‐Table5.docx.

**Supporting File 8**: advs78577‐sup‐0008‐SuppMat.pdf.

**Supporting File 9**: advs78577‐sup‐0009‐SuppMat.docx.

## Data Availability

The datasets used and/or analyzed during the current study are available from the corresponding author on reasonable request. The public datasets analyzed in this study were obtained from the following sources: TCGA (Colorectal Adenocarcinoma, PanCancer Atlas), Colorectal Adenocarcinoma (TCGA, Nature 2012), Colon Cancer (CPTAC‐2 Prospective, Cell 2019), Colorectal Adenocarcinoma (TCGA, Firehose Legacy), GSE37283, GSE40967, GSE231559, GSE132465, GSE221575, GSE13294, GSE39084, GSE44988, GSE44904, GSE46200, GSE43338, GSE64658, GSE4183, GSE39582, GSE3629, GSE87466, GSE47908, GSE38713, GSE92415, GSE11223, GSE107499, GSE66407, GSE14333, GSE179285, GSE87473, GSE10191, GSE48958, GSE13367, GSE36807, GSE17536, GSE9348, GSE10616, GSE212849, GSE188711, and GSE72970. The RAW data from the mouse single‐cell RNA sequencing are available for download at GitHub (https://github.com/Fancy1123611/305Lab/tree/main). Functional analysis of the DEGs was conducted using the DAVID bioinformatics database for pathway enrichment analysis (KEGG) (http://david.ncifcrf.gov/tools.jsp). All detailed code for this study is available on GitHub (https://github.com/Fancy1123611/305Lab/tree/main).

## References

[1] J. Terzić , S. Grivennikov , E. Karin , and M. Karin , “Inflammation and Colon Cancer,” Gastroenterology 138, no. 6 (2010): 2101–2114.e5, 10.1053/j.gastro.2010.01.058. 20420949

[2] X. Liang , X. Li , R. Wu , et al., “Breaking the Tumor Chronic Inflammation Balance With a Programmable Release and Multi‐Stimulation Engineering Scaffold for Potent Immunotherapy,” Advanced Science 11, no. 28 (2024): 2401377, 10.1002/advs.202401377. PMC11267263 38760901

[3] M. Schmitt and F. R. Greten , “The Inflammatory Pathogenesis of Colorectal Cancer,” Nature Reviews Immunology 21, no. 10 (2021): 653–667, 10.1038/s41577-021-00534-x. 33911231

[4] S. I. Grivennikov , “Inflammation and Colorectal Cancer: Colitis‐Associated Neoplasia,” Seminars in Immunopathology 35, no. 2 (2013): 229–244, 10.1007/s00281-012-0352-6. PMC3568220 23161445

[5] X. Liu , L. Yin , S. Shen , and Y. Hou , “Inflammation and Cancer: Paradoxical Roles in Tumorigenesis and Implications in Immunotherapies,” Genes & Diseases 10, no. 1 (2023): 151–164, 10.1016/j.gendis.2021.09.006. PMC10066281 37013041

[6] S. Shalapour and M. Karin , “Immunity, Inflammation, and Cancer: an Eternal Fight Between Good and Evil,” Journal of Clinical Investigation 125, no. 9 (2015): 3347–3355, 10.1172/JCI80007. PMC4588298 26325032

[7] R. De Matteis , M. B. Flak , M. Gonzalez‐Nunez , et al., “Aspirin Activates Resolution Pathways to Reprogram T Cell and Macrophage Responses in Colitis‐Associated Colorectal Cancer,” Science Advances 8 (2022): abl5420, 10.1126/sciadv.abl5420. PMC8809687 35108049

[8] T. Hirano , D. Hirayama , K. Wagatsuma , T. Yamakawa , Y. Yokoyama , and H. Nakase , “Immunological Mechanisms in Inflammation‐Associated Colon Carcinogenesis,” International Journal of Molecular Sciences 21, no. 9 (2020): 3062, 10.3390/ijms21093062. PMC7247693 32357539

[9] A. Abaurrea , A. M. Araujo , and M. M. Caffarel , “The Role of the IL‐6 Cytokine Family in Epithelial–Mesenchymal Plasticity in Cancer Progression,” International Journal of Molecular Sciences 22, no. 15 (2021): 8334, 10.3390/ijms22158334. PMC8347315 34361105

[10] J. Noh , C. You , K. Kang , and K. Kang , “Activation of OSM‐STAT3 Epigenetically Regulates Tumor‐Promoting Transcriptional Programs in Cervical Cancer,” Cancers 14, no. 24 (2022): 6090, 10.3390/cancers14246090. PMC9775986 36551576

[11] W. Pu , C. Ma , B. Wang , W. Zhu , and H. Chen , “The “Heater” of “Cold” Tumors–Blocking IL‐6,” Advanced Biology 8, no. 7 (2024): 2300587, 10.1002/adbi.202300587. 38773937

[12] J. Bromberg and T. C. Wang , “Inflammation and Cancer: IL‐6 and STAT3 Complete the Link,” Cancer Cell 15, no. 2 (2009): 79–80, 10.1016/j.ccr.2009.01.009. PMC3684978 19185839

[13] S. Grivennikov , E. Karin , J. Terzic , et al., “IL‐6 and Stat3 Are Required for Survival of Intestinal Epithelial Cells and Development of Colitis‐Associated Cancer,” Cancer Cell 15, no. 2 (2009): 103–113, 10.1016/j.ccr.2009.01.001. PMC2667107 19185845

[14] J. Bollrath , T. J. Phesse , V. A. von Burstin , et al., “Gp130‐Mediated Stat3 Activation in Enterocytes Regulates Cell Survival and Cell‐Cycle Progression During Colitis‐Associated Tumorigenesis,” Cancer Cell 15, no. 2 (2009): 91–102, 10.1016/j.ccr.2009.01.002. 19185844

[15] Y. Lin , Z. He , J. Ye , et al., “Progress in Understanding the IL‐6/STAT3 Pathway in Colorectal Cancer,” OncoTargets and therapy 13 (2020): 13023–13032, 10.2147/OTT.S278013. PMC7762435 33376351

[16] A. Goenka , F. Khan , B. Verma , et al., “Tumor Microenvironment Signaling and Therapeutics in Cancer Progression,” Cancer Communications 43, no. 5 (2023): 525–561, 10.1002/cac2.12416. PMC10174093 37005490

[17] J. Zhu , B. Xu , R. Hu , et al., “Reprogramming Tumor‐Associated Macrophages via Targeted NAT10 Inhibition to Enhance Colorectal Cancer Immunotherapy,” Advanced Science 13 (2026): 10854, 10.1002/advs.202510854. PMC12786336 41144699

[18] N. Kumari and S. H. Choi , “Tumor‐Associated Macrophages in Cancer: Recent Advancements in Cancer Nanoimmunotherapies,” Journal of Experimental & Clinical Cancer Research 41, no. 1 (2022): 68, 10.1186/s13046-022-02272-x. PMC8857848 35183252

[19] Y. Yang , S. Li , K. K. W. To , S. Zhu , F. Wang , and L. Fu , “Tumor‐Associated Macrophages Remodel the Suppressive Tumor Immune Microenvironment and Targeted Therapy for Immunotherapy,” Journal of Experimental & Clinical Cancer Research 44, no. 1 (2025): 145, 10.1186/s13046-025-03377-9. PMC12083052 40380196

[20] E. Timosenko and D. I. Gabrilovich , “Regulating the Fate of Tumor‐Associated Macrophages,” Cancer Cell 43, no. 7 (2025): 1191–1193, 10.1016/j.ccell.2025.04.006. 40345187

[21] M. Rakina , I. Larionova , and J. Kzhyshkowska , “Macrophage Diversity in Human Cancers: New Insight Provided by Single‐Cell Resolution and Spatial Context,” Heliyon 10 (2024): e28332, 10.1016/j.heliyon.2024.e28332. PMC10988020 38571605

[22] K. Inagaki , S. Kunisho , H. Takigawa , et al., “Role of Tumor‐Associated Macrophages at the Invasive Front In Human Colorectal Cancer Progression,” Cancer Science 112, no. 7 (2021): 2692–2704, 10.1111/cas.14940. PMC8253270 33964093

[23] G. Yu , L.‐G. Wang , Y. Han , and Q.‐Y. He , “clusterProfiler: an R Package for Comparing Biological Themes Among Gene Clusters,” OMICS : a Journal of Integrative Biology 16 (2012): 284–287, 10.1089/omi.2011.0118. PMC3339379 22455463

[24] H. Wang , L. Zhang , H. Liu , et al., “PDZK1 Confers Sensitivity to Sunitinib in Clear Cell Renal Cell Carcinoma by Suppressing the PDGFR‐β Pathway,” British Journal of Cancer 131, no. 2 (2024): 347–360, 10.1038/s41416-024-02725-4. PMC11263541 38822145

[25] C. Zhao , T. Tao , L. Yang , et al., “Loss of PDZK1 Expression Activates PI3K/AKT Signaling via PTEN Phosphorylation in Gastric Cancer,” Cancer Letters 453 (2019): 107–121, 10.1016/j.canlet.2019.03.043. 30930234

[26] Y. Hao , S. Hao , E. Andersen‐Nissen , et al., “Integrated Analysis of Multimodal Single‐Cell Data,” Cell 184, no. 13 (2021): 3573–3587.e29, 10.1016/j.cell.2021.04.048. PMC8238499 34062119

[27] A. Butler , P. Hoffman , P. Smibert , E. Papalexi , and R. Satija , “Integrating Single‐Cell Transcriptomic Data Across Different Conditions, Technologies, and Species,” Nature biotechnology 36, no. 5 (2018): 411–420, 10.1038/nbt.4096. PMC6700744 29608179

[28] A. L. Ji , A. J. Rubin , K. Thrane , et al., “Multimodal Analysis of Composition and Spatial Architecture in Human Squamous Cell Carcinoma,” Cell 182 (2020): 497–514, 10.1016/j.cell.2020.05.039. PMC7391009 32579974

[29] X. Qiu , Q. Mao , Y. Tang , et al., “Reversed Graph Embedding Resolves Complex Single‐Cell Trajectories,” Nature Methods 14, no. 10 (2017): 979–982, 10.1038/nmeth.4402. PMC5764547 28825705

[30] C. Trapnell , D. Cacchiarelli , J. Grimsby , et al., “The Dynamics and Regulators of Cell Fate Decisions Are Revealed by Pseudotemporal Ordering of Single Cells,” Nature Biotechnology 32, no. 4 (2014): 381–386, 10.1038/nbt.2859. PMC4122333 24658644

[31] A. Tovchigrechko and I. A. Vakser , “GRAMM‐X public web server for Protein‐Protein Docking,” Nucleic Acids Research 34 (2006): W310–W314, 10.1093/nar/gkl206. PMC1538913 16845016

[32] M. Zhang , X. Li , Q. Zhang , J. Yang , and G. Liu , “Roles of Macrophages on Ulcerative Colitis and Colitis‐Associated Colorectal Cancer,” Frontiers in Immunology 14 (2023): 1103617, 10.3389/fimmu.2023.1103617. PMC10062481 37006260

[33] A. E. Shin , Y. Tesfagiorgis , F. Larsen , et al., “F4/80^+^Ly6Chigh Macrophages Lead to Cell Plasticity and Cancer Initiation in Colitis,” Gastroenterology 164, no. 4 (2023): 593–609.e13, 10.1053/j.gastro.2023.01.002. PMC10038892 36634827

[34] H. Yang , Q. Zhang , M. Xu , et al., “CCL2‐CCR2 Axis Recruits Tumor Associated Macrophages to Induce Immune Evasion Through PD‐1 Signaling in Esophageal Carcinogenesis,” Molecular Cancer 19, no. 1 (2020): 41, 10.1186/s12943-020-01165-x. PMC7045401 32103760

[35] R. L. Philips , Y. Wang , H. Cheon , et al., “The JAK‐STAT Pathway at 30: Much Learned, Much More to Do,” Cell 185, no. 21 (2022): 3857–3876, 10.1016/j.cell.2022.09.023. PMC9815833 36240739

[36] M. Castro‐Cruz , F. Lembo , J.‐P. Borg , G. Travé , R. Vincentelli , and P. Zimmermann , “The Human PDZome 2.0: Characterization of a New Resource to Test for PDZ Interactions by Yeast Two‐Hybrid,” Membranes 13, no. 8 (2023): 737, 10.3390/membranes13080737. PMC10456741 37623798

[37] Y. Belo , Z. Mielko , H. Nudelman , et al., “Unexpected Implications of STAT3 Acetylation Revealed by Genetic Encoding of Acetyl‐Lysine,” Biochimica et Biophysica Acta (BBA)—General Subjects 1863, no. 9 (2019): 1343–1350, 10.1016/j.bbagen.2019.05.019. PMC6625855 31170499

[38] I. D. Pogozheva , S. Cherepanov , S.‐J. Park , M. Raghavan , W. Im , and A. L. Lomize , “Structural Modeling of Cytokine‐Receptor‐JAK2 Signaling Complexes Using AlphaFold Multimer,” Journal of Chemical Information and Modeling 63, no. 18 (2023): 5874–5895, 10.1021/acs.jcim.3c00926. PMC11791896 37694948

[39] Y. Jin , Y. Kang , M. Wang , et al., “Targeting Polarized Phenotype of Microglia via IL6/JAK2/STAT3 Signaling to Reduce NSCLC Brain Metastasis,” Signal Transduction and Targeted Therapy 7, no. 1 (2022): 52, 10.1038/s41392-022-00872-9. PMC8864012 35194016

[40] Y. Hu , Z. Dong , and K. Liu , “Unraveling the Complexity of STAT3 in Cancer: Molecular Understanding and Drug Discovery,” Journal of Experimental & Clinical Cancer Research 43, no. 1 (2024): 23, 10.1186/s13046-024-02949-5. PMC10799433 38245798

[41] S. Zou , Q. Tong , B. Liu , W. Huang , Y. Tian , and X. Fu , “Targeting STAT3 in Cancer Immunotherapy,” Molecular Cancer 19, no. 1 (2020): 145, 10.1186/s12943-020-01258-7. PMC7513516 32972405

[42] J. A. Eaden , K. R. Abrams , and J. F. Mayberry , “The Risk of Colorectal Cancer in Ulcerative Colitis: a Meta‐Analysis,” Gut 48, no. 4 (2001): 526–535, 10.1136/gut.48.4.526. PMC1728259 11247898

[43] Q. Ma , Y. Zhang , R. Meng , et al., “MAGI3 Suppresses Glioma Cell Proliferation via Upregulation of PTEN Expression,” Biomedical and Environmental Sciences 28 (2015): 502–509, 10.3967/bes2015.072. 26248734

[44] Q. Ma , Y. Yang , D. Feng , et al., “MAGI3 Negatively Regulates Wnt/β‐Catenin Signaling and Suppresses Malignant Phenotypes of Glioma Cells,” Oncotarget 6, no. 34 (2015): 35851–35865, 10.18632/oncotarget.5323. PMC4742146 26452219

[45] H. Wang , Y. Chen , Y. Yang , et al., “MAGI3 Enhances Sensitivity to Sunitinib in Renal Cell Carcinoma by Suppressing the MAS/ERK Axis and Serves as a Prognostic Marker,” Cell Death & Disease 16, no. 1 (2025): 102, 10.1038/s41419-025-07427-0. PMC11830799 39956807

[46] Z. Yang , H. Liu , R. Song , et al., “Reduced MAGI3 Level by HPV18E6 Contributes to Wnt/β‐Catenin Signaling Activation and Cervical Cancer Progression,” FEBS Open Bio 11, no. 11 (2021): 3051–3062, 10.1002/2211-5463.13298. PMC8564337 34510826

[47] T. Tanaka , Y. Yamamoto , R. Muromoto , et al., “PDLIM2 Inhibits T Helper 17 Cell Development and Granulomatous Inflammation Through Degradation of STAT3,” Science Signaling 4, no. 202 (2011): ra85, 10.1126/scisignal.2001637. 22155789

[48] S. Hwang , J. Park , S.‐Y. Koo , et al., “The Ubiquitin Ligase Pellino1 Targets STAT3 to Regulate Macrophage‐Mediated Inflammation and Tumor Development,” Nature Communications 16, no. 1 (2025): 1256, 10.1038/s41467-025-56440-6. PMC11787384 39893188

[49] L. Jia , Y. Wang , and C. Wang , “circFAT1 Promotes Cancer Stemness and Immune Evasion by Promoting STAT3 Activation,” Advanced Science 8 (2021): 2003376, 10.1002/advs.202003376. PMC8261519 34258151

[50] H. Wang , W. Yang , Q. Qin , et al., “E3 Ubiquitin Ligase MAGI3 Degrades C‐Myc and Acts as a Predictor for Chemotherapy Response in Colorectal Cancer,” Molecular Cancer 21, no. 1 (2022): 151, 10.1186/s12943-022-01622-9. PMC9306183 35864508

[51] D. Gyori , E. L. Lim , F. M. Grant , et al., “Compensation between CSF1R^+^ macrophages and Foxp3^+^Treg Cells Drives Resistance to Tumor Immunotherapy,” JCI Insight 3, no. 11 (2018): e120631, 10.1172/jci.insight.120631. PMC6124419 29875321

[52] A. R. Razak , J. M. Cleary , V. Moreno , et al., “Safety and Efficacy of AMG 820, an Anti‐Colony‐Stimulating Factor 1 Receptor Antibody, in Combination With Pembrolizumab in Adults With Advanced Solid Tumors,” Journal for Immunotherapy of Cancer 8, no. 2 (2020): 001006, 10.1136/jitc-2020-001006. PMC7552843 33046621

[53] S. K. Sandhu , K. Papadopoulos , P. C. Fong , et al., “A First‐in‐Human, First‐in‐Class, Phase I Study of Carlumab (CNTO 888), a Human Monoclonal Antibody Against CC‐Chemokine Ligand 2 in Patients With Solid Tumors,” Cancer Chemotherapy and Pharmacology 71, no. 4 (2013): 1041–1050, 10.1007/s00280-013-2099-8. 23385782

[54] I. Brana , A. Calles , P. M. LoRusso , et al., “Carlumab, an Anti‐C‐C Chemokine Ligand 2 Monoclonal Antibody, in Combination With Four Chemotherapy Regimens for the Treatment of Patients With Solid Tumors: an Open‐Label, Multicenter Phase 1b Study,” Targeted Oncology 10, no. 1 (2015): 111–123, 10.1007/s11523-014-0320-2. 24928772

[55] K. J. Pienta , J.‐P. Machiels , D. Schrijvers , et al., “Phase 2 Study of Carlumab (CNTO 888), a Human Monoclonal Antibody Against CC‐Chemokine Ligand 2 (CCL2), in Metastatic Castration‐Resistant Prostate Cancer,” Investigational New Drugs 31, no. 3 (2013): 760–768, 10.1007/s10637-012-9869-8. 22907596

