# Supplementary material for: MAGI3 Acts as an Intestinal Epithelial Gatekeeper in Inflammation‐Associated Colorectal Cancer by Suppressing the STAT3‐CCL2 Axis and TAM‐Mediated Immunosuppression
Source: Adv Sci (Weinh). 2026 Oct 31:e78577. Online ahead of print. doi: 10.1002/advs.78577 (PMC13723711; doi:10.1002/advs.78577)
Supplement: Supplementary file 2 [file ADVS-9999-e78577-s001.docx]

**Supplemental information**

**Fig. S1 Integrated transcriptomic analyses and single-cell sequencing identify epithelial MAGI3 as a key regulator of the inflammation-to-cancer transition.**

**A-D.** Quality control and batch-effect correction of integrated transcriptomic datasets (GSE3629, GSE37283, and GSE47908) for downstream analysis.
**A.** Hierarchical clustering of samples to identify potential outliers.
**B**, **C.** Boxplots of the three datasets before **(B)** and after batch-effect correction **(C)**.
**D.** Principal component analysis (PCA) of the integrated gene-expression datasets after batch-effect correction, colored by disease group **(left)** and dataset source **(right)**.

**E.** Single-cell RNA-seq analysis of colorectal cancer tissues (GSE231559): dot plot of canonical marker genes used for cell-type annotation **(left)**, UMAP visualization of single-cell clusters **(middle)**, and annotated UMAP colored by assigned cell types **(right)**.

**F.** Feature plots showing UMAP visualizations of the 15 intersecting genes at single-cell resolution. Color intensity indicates normalized gene expression levels.

**G.** Proportion of MAGI3-positive cells across major cell populations in the GSE231559 single-cell RNA-seq dataset. Percentages are indicated above the bars.

**H.** Heatmap showing MAGI3 expression and canonical marker genes across the annotated cell populations in GSE231559.

**Fig. S2 scRNA-seq analyses show preferential MAGI3 expression in colorectal epithelial cells.**

**A-D.** scRNA-seq analysis of the GSE132465 CRC dataset.

**A.** UMAP visualization of the major annotated cell populations.

**B.** Feature plot showing the distribution of MAGI3 expression across cell populations.

**C.** Heatmap showing MAGI3 expression and canonical marker genes used for cell-type annotation.

**D.** Proportion of MAGI3-positive cells across the major cell populations. Percentages are indicated above the bars.

**E-H.** Validation in the independent GSE221575 CRC scRNA-seq dataset.

**E.** UMAP visualization of the major annotated cell populations.

**F.** Feature plot showing the distribution of MAGI3 expression across cell populations.

**G.** Heatmap showing MAGI3 expression and canonical marker genes used for cell-type annotation.

**H.** Proportion of MAGI3-positive cells across the major cell populations. Percentages are indicated above the bars.

**Fig. S3 MAGI3 downregulation in ulcerative colitis and marker-based annotation of the GSE188711 CRC scRNA-seq dataset.**

**A.** MAGI3 mRNA expression is consistently lower in patients with UC than in controls across multiple datasets (GSE48958, GSE10191, GSE87466, GSE87473, and GSE179285). Statistical significance was determined using the Mann–Whitney U test for all datasets.

**B.** MAGI3 mRNA expression in paired non-lesional and lesional tissues from patients with UC (GSE107499; n = 12 pairs; paired t-test).

**C.** scRNA-seq analysis of colorectal cancer tissues (GSE188711): dot plots showing the expression of canonical cell-type markers **(left)** and t-SNE visualization of cell clusters **(right)**.
All quantitative data are presented as mean ± SEM. **P* < 0.05, ***P* < 0.01, ****P* < 0.001, *****P < 0.0001; ns, not significant.*

**Fig. S4 Effects of DSS-induced colitis on colon length, spleen index, disease activity, and body weight change.**
**A.** Schematic diagram of the experimental timeline showing the DSS treatment regimen (2% DSS for 7 days, followed by 1% DSS for 10 days, and 2% DSS for 7 days).
**B.** Representative images of colons and spleens from control and DSS-treated mice at the experimental endpoint (n = 3 control mice and n = 5 DSS-treated mice).

**C.** Measurement of colon length in control mice (red, n = 3) and DSS-treated mice (blue, n = 5), showing a significant reduction in colon length after DSS treatment. Data are presented as mean ± SEM. *****P* < 0.0001.
**D.** Spleen index (spleen weight as a percentage of body weight) in control mice (n = 3) and DSS-treated mice (n = 5), showing a significant increase after DSS treatment. Data are presented as mean ± SEM. ***P* < 0.01.
**E.** Disease activity index (DAI) scores over time in control mice (n = 3) and DSS-treated mice (n = 5), indicating a significant increase in disease severity after DSS treatment. Statistical significance was determined by two-way ANOVA with Tukey’s multiple-comparisons test. Data are presented as mean ± SEM. ***P* < 0.01, *****P* < 0.0001; *ns*, not significant.

**F.** Relative body weight over time in control mice (n = 3) and DSS-treated mice (n = 5), normalized to day 0. Statistical significance was determined by two-way ANOVA with Tukey’s multiple-comparisons test. Data are presented as mean ± SEM. ***P* < 0.01, *****P* < 0.0001; *ns*, not significant.

**G.** Representative H&E-stained Swiss-roll sections of the colon from control mice and mice with DSS-induced chronic colitis.

**Fig. S5 MAGI3 expression is decreased in AOM/DSS mice.**

**A-E.** Scatter plots of MAGI3 mRNA expression in control and AOM/DSS-treated mice across GEO datasets. Data are presented as mean ± SEM.

**A.** GSE44988: n = 3 for both control and AOM/DSS groups. **P* < 0.05.

**B.** GSE44904: n = 3 for both control and AOM/DSS groups. **P* < 0.05.

**C.** GSE46200: n = 14 for control, n = 16 for AOM/DSS. ***P* < 0.01.

**D.** GSE43338: n = 2 for control, n = 4 for AOM/DSS. *****P* < 0.0001.

**E.** GSE64658: n = 12 for control, n = 16 for AOM/DSS. **P* < 0.05.

**Fig. S6 Generation, validation, and baseline phenotypic characterization of *Magi3*-knockout mice.**

**A.** Representative images of WT and *Magi3^⁻/⁻^* mice.

**B-C.** Generation and validation of *Magi3*-knockout mice using CRISPR/Cas9-mediated genome editing.

**B.** Schematic representation of the *Magi3* wild-type allele and the targeted allele after Cas9/sgRNA-mediated deletion.

**C.** Genotyping PCR confirmation of *Magi3* deletion in *Magi3^⁻/⁻^* mice.

**D-F.** Baseline body weight **(D)**, representative colon images and colon length **(E)**, and representative spleen images and spleen index **(F)** in WT and *Magi3^-/-^* mice (n = 3 mice per group). Statistical significance was assessed using the Mann–Whitney U test. Data are presented as mean ± SEM. *ns*, not significant.

**G.** Representative H&E-stained sections of the colon, lung, liver, kidney, and spleen from WT and *Magi3^⁻/⁻^* mice. No apparent histological abnormalities were observed in *Magi3*-deficient mice. Scale bars, 2 mm (overview images) and 200 μm (enlarged images).

**Fig. S7 Broader immune cell profiling in AOM/DSS-induced colorectal tumors from WT and *Magi3*-deficient mice.**

Representative immunohistochemical staining and corresponding H-score quantification of CD45R/B220^+^ B cells, NKp46^+^ NK cells, CD8^+^ cytotoxic T cells, CD4^+^ T cells, and FOXP3^+^ regulatory T cells in colorectal tumor tissues from WT and *Magi3^-/-^* mice. n = 5 mice per group. Data are presented as the mean ± SEM. Statistical significance was assessed using the Mann–Whitney U test. **P* < 0.05; *ns*, not significant.

**Fig. S8 MAGI3 and CCL2 mRNA expression levels are negatively correlated at the transcriptional level in patients with UC and CRC cohorts.**

**A.** Correlation heatmap of MAGI3 with chemokine expression in the GSE39582 dataset.

**B-F.** Pearson correlations between MAGI3 mRNA expression and CCL8 **(B)**, CXCL10 **(C)**, CXCL9 **(D)**, CCL5 **(E)**, or CCL2 **(F)** mRNA expression in the GSE17536 CRC cohort (n = 177).

**G-N.** Pearson correlations between MAGI3 and CCL2 mRNA expression in GSE38713 (**G**, n = 30), GSE36807 (**H**, n = 15), TCGA (**I**, n = 594), GSE107499 (**J**, n = 119), GSE13367 (**K**, n = 34), GSE10616 (**L**, n = 41), GSE4183 (**M**, n = 15), and GSE72970 (**N**, n = 124).

**Fig. S9 MAGI3 expression is specifically and inversely correlated with STAT3 pathway activity among STAT family members.**
**A-F.** ssGSEA scores for STAT1-STAT6 signaling pathways in MAGI3-low vs. MAGI3-high subgroups in the GSE81980 cohort (CRC, n = 149). Only the STAT3 pathway score was significantly higher in the MAGI3-low group than in the MAGI3-high group **(C)**. Statistical significance was assessed using a two-tailed unpaired Student's t-test **(A–D and F)** or the Mann–Whitney U test **(E)**. The MAGI3-low and MAGI3-high groups included 75 and 74 patients, respectively. Data are presented as mean ± SEM. * *P*< 0.05; *ns*, not significant.

**Fig. S10 MAGI3 and STAT3 mRNA expression levels are not significantly correlated in the analyzed GEO cohorts**.

**A–G.** Correlation analyses between MAGI3 and STAT3 mRNA expression levels in GSE38713 (**A**; UC, n = 30), GSE36807 (**B**; UC, n = 15), GSE10616 (**C**; UC, n = 10), GSE13367 (**D**; UC, n = 34), the CRC subset of GSE4183 (**E**; n = 15), GSE37283 (**F**; UC, n = 15), and the IBD subset of GSE4183 (**G**; n = 15). Panels **E** and **G** show separate analyses of distinct CRC and IBD sample groups within the same GSE4183 dataset.

**Fig. S****11 MAGI3 overexpression does not significantly affect STAT3 protein stability in NCM460 cells.**

**A.** The effect of MAGI3 on the half-life of STAT3 was assessed by western blotting in NCM460 cells treated with cycloheximide (CHX). n = 3 independent biological replicates.

**B.** The protein half-life curves were obtained by quantifying three independent experiments. Data are presented as mean ± SEM. Statistical significance was assessed by two-way ANOVA with Šidák’s multiple comparisons test. *ns*, not significant.

**Fig. S12 CCL2 neutralization attenuates the tumor-promoting effects of MAGI3 depletion and suppresses tumor growth.**

**A.** Representative images of excised CT26 tumors from mice bearing CT26-sgNC or CT26-sgMAGI3 tumors and treated with either isotype control IgG or an anti-CCL2 neutralizing antibody. Tumors were harvested at the experimental endpoint.

**B.** Quantification of tumor weights in the indicated experimental groups (n = 5 mice per group). Data are presented as mean ± SEM. Statistical significance was determined by one-way ANOVA followed by Tukey’s multiple-comparisons test. *****P* < 0.0001; *ns*, not significant.

**C.** Tumor growth curves of mice bearing CT26-sgNC or CT26-sgMAGI3 tumors and treated with isotype control IgG or a CCL2-neutralizing antibody. Tumor volumes were measured at the indicated time points. Data are presented as mean ± SEM (n = 5 mice per group). Statistical analysis was performed using two-way ANOVA with Šidák’s multiple comparisons test. *****P* < 0.0001; *ns*, not significant.

**D.** Representative immunohistochemical staining and quantification of MAGI3, p-STAT3^Y705^, and CD206 in tumor tissues from the indicated groups. Positive staining is shown in brown, and enlarged views of the boxed regions are shown below each overview image. Scale bars, 60 μm (overview images) and 20 μm (enlarged images). H-scores were calculated based on staining intensity and the percentage of positive cells. Data are presented as mean ± SEM (n = 5 mice per group). Statistical significance was assessed using the Mann–Whitney U test. ***P* < 0.01; *ns*, not significant.

**Fig. S13 MAGI3 expression selectively and inversely correlates with JAK2-mediated signaling.**

**A-D.** GSEA plots showing enrichment of downstream gene sets for JAK2 **(A)**, JAK1 **(B)**, TYK2 **(C)**, and JAK3 **(D)** between MAGI3-low and MAGI3-high subgroups in the GSE81980 cohort (CRC, n = 149). n = 75 patients for the Low MAGI3 group, n = 74 for the High MAGI3 group.

**Fig. S14** **Molecular docking and structural modeling of MAGI3-mediated inhibition of STAT3 phosphorylation.**

**A.** Crystal structure of a phosphorylated STAT3 dimer–DNA complex (PDB ID: 6QHD).

**B.** Crystal structure of a STAT3 monomer.

**C.** Magnified view of the boxed region in panel **B**. The phosphotyrosine-binding (pY) pocket of the SH2 domain, which is critical for STAT3 activation, is shown in red.

**D.** Crystal structure of the MAGI3 protein, with the PDZ1 domain highlighted.

**E-F.** Molecular docking models of the STAT3/MAGI3-PDZ1 complex indicate that binding of MAGI3-PDZ1 to the STAT3 PBM obstructs the pY-binding pocket of STAT3 **(E)**. The right panels show magnified views of the boxed regions in the corresponding left panels **(F)**.

**G-H.** Molecular modeling workflow **(G)** and schematic illustration **(H)** of MAGI3-mediated inhibition of STAT3 phosphorylation.

**Fig. S15 *Magi3* knockout enhances STAT3 activation and M2-like macrophage infiltration in AOM/DSS-induced colorectal tumors.**

**A-B.** Representative images were shown to indicate the expression of MAGI3, p-STAT3^Y705^, CD206, CD163, CCL2 and Ki67 in different groups (n = 4 mice per group) determined by immunohistochemistry assay **(A)**. Scale bar, 100 μm (left). Scale bar, 60 μm (right). Quantification of H-scores for MAGI3, p-STAT3^Y705^, CD206, CD163, CCL2, and Ki67 expression in tumor tissues **(B,** n = 4 mice per group**)**. Statistical significance was determined using Mann–Whitney U test. Data are presented as mean ± SEM. **P*<0.05, ***P*<0.01, ****P* < 0.001, *****P* < 0.0001.

**C.** Western blot analysis of MAGI3, total STAT3, and p-STAT3^Y705^ in tumor tissues from AOM/DSS-treated WT and *Magi3*-knockout mice (n = 4 mice per group). β-actin was used as the loading control.

**Fig. S16 Nomograms predicting overall survival (OS) and progression-free survival (PFS) in patients with colorectal cancer.**

**A-B.** Nomograms for predicting OS **(A)** and PFS **(B)** based on pathologic T stage, CEA level, MAGI3 expression, and CCL2 expression. These nomograms were constructed using Cox proportional hazards regression models based on data from a cohort of n = 649 patients. The total points are calculated by summing the individual points assigned to each variable, and these correspond to the linear predictor used to estimate the 1-year, 3-year, and 5-year survival probabilities.

**Fig. S17 GSVA and GSEA analyses of c-Myc and STAT3 pathway signatures across CMS1-CMS4 CRC subtypes stratified by MAGI3 expression.**

**A.** Heatmap showing gene-expression profiles of CRC tumor samples from the TCGA cohort. Tumors were assigned to one of the four consensus molecular subtypes (CMS1, CMS2, CMS3, or CMS4).

**B-C.** Comparison of GSVA scores for c-Myc **(B)** and STAT3 **(C)** pathway signatures across CMS subtypes (CMS1: n = 96, CMS2: n = 169, CMS3: n = 96, CMS4: n = 172). Statistical analysis was performed using the Kruskal-Wallis test followed by post hoc multiple comparisons. Data are presented as the mean ± SEM. **P* < 0.05, *****P* < 0.0001, *ns*, not significant.

**D.** GSEA of STAT3-**(left)** and c-Myc-related signatures**(right)** in patients with CMS2 tumors stratified into MAGI3-high (n = 85) and MAGI3-low (n = 84) groups.

**E.** GSEA of the STAT3- **(left)** and c-Myc-related signatures **(right)** in a pooled group of patients with CMS1 or CMS4 tumors, stratified into MAGI3-low (n = 134) and MAGI3-high (n = 134) groups.

**Fig. S18 Inflammation-dependent association of MAGI3 with STAT3 signaling and tumor microenvironmental features in CRC.**

**A.** Comparison of inflammation-related GSVA scores between the high-inflammation (n = 53) and low-inflammation (n = 53) subgroups in the CPTAC cohort. *****P* < 0.0001.

**B-C.** GSEA of IL-6/JAK/STAT3 signaling **(left)** and c-Myc target gene signatures **(right)** within the high-inflammation **(B)** and low-inflammation **(C)** subgroups of the CPTAC cohort. Each subgroup was stratified into MAGI3-low (n = 27) and MAGI3-high (n = 26) groups.

**D-E.** GSEA of apoptosis **(left)**, epithelial–mesenchymal transition (EMT; **middle**), and M2-like macrophage gene signatures **(right)** within the high-inflammation **(D)** and low-inflammation **(E)** subgroups of the CPTAC cohort, comparing MAGI3-low (n = 27) and MAGI3-high (n = 26) tumors.

**Fig. S19 MAGI3 mRNA expression does not differ significantly by MSS/MSI-H status, CIN status, or *KRAS*, *BRAF*^V600E^, or *TP53* mutation status.**

**A-D.** Scatter plots depict the differences in MAGI3 expression levels between MSS and MSI-H patients in the TCGA (n = 313 for MSS, n = 52 for MSI-H), GSE13294 (n = 77 for MSS, n = 78 for MSI-H), GSE18088 (n = 34 for MSS, n = 19 for MSI-H), and GSE39084 (n = 54 for MSS, n = 16 for MSI-H) cohorts.

**E.** Scatter plot illustrates the differences in MAGI3 expression levels between CIN (-) and CIN (+) patients in the GSE39582 (n = 112 for CIN (-), n = 369 for CIN (+)) cohort.

**F-G.** Scatter plots show the differences in MAGI3 expression levels between KRAS wild-type and KRAS-mutant patients in the GSE39582 (n = 328 for KRAS wild-type, n = 217 for KRAS- mutation) and GSE39084 (n = 29 for KRAS wild-type, n = 18 for KRAS-mutation) cohorts.

**H-I.** Scatter plots present the differences in MAGI3 expression levels between BRAF wild-type and BRAF-mutant patients in the GSE39582 (n = 461 for BRAF wild-type, n = 51 for BRAF-mutation) and GSE39084 (n = 62 for BRAF wild-type, n = 8 for BRAF-mutation) cohorts.

**J-K.** Scatter plots display the differences in MAGI3 expression levels between TP53 wild-type and TP53-mutant patients in the GSE39582 (n = 161 for TP53 wild-type, n = 190 for TP53- mutation) and GSE39084 (n = 39 for TP53 wild-type, n = 31 for TP53-mutation) cohorts.

The Mann–Whitney U test was used for panels **B** and **E**, whereas a two-tailed unpaired Student's t-test was used for all other panels. Data are presented as mean ± SEM. *ns*, not significant.

**Fig. S20 MAGI3-low tumors with concurrent high inflammation represent a STAT3-hyperactive, chemotherapy-resistant subtype.**

**A.** Proportions of chemotherapy-resistant and chemotherapy-sensitive patients in the MAGI3-high (n = 51) and MAGI3-low expression (n = 51) groups from the GSE72970 cohort. Statistical significance was assessed using the chi-square test. * *P* < 0.05.

**B.** Inflammation scores in MAGI3-low patients stratified into high-inflammation (n = 38) and low-inflammation (n = 13) subgroups in the GSE72970 cohort. Inflammation scores were derived from COX-2 expression levels. Data are presented as mean ± SEM. *****P* < 0.0001.

**C-D.** GSVA scores for STAT3 **(C)** and c-Myc **(D)** pathway signatures in MAGI3-low patients stratified into high-inflammation (n = 38) and low-inflammation (n = 13) subgroups in GSE72970, with inflammation scores based on COX-2 expression. Data are presented as mean ± SEM. ***P* < 0.01, *ns*, not significant.

**E.** Stratification of MAGI3-low patients in the GSE72970 cohort into high-inflammation (n = 38) and low-inflammation (n = 13) subgroups, showing proportions of chemotherapy-resistant and chemotherapy-sensitive cases. Statistical significance was assessed using Fisher’s exact test. **P* < 0.05.

**F.** Proportions of chemotherapy-resistant and chemotherapy-sensitive cases in high- and low-inflammation subgroups (n = 27 each) from an independent 230-patient CRC cohort. Statistical significance was assessed using Fisher’s exact test. * *P* < 0.05.

**G.** Kaplan–Meier analysis of recurrence-free survival (RFS) in high- and low-inflammation subgroups (n = 27 each) from the independent 230-patient CRC cohort. **P* < 0.05 (log-rank test).
