# Supplementary material for: MAGI3 Acts as an Intestinal Epithelial Gatekeeper in Inflammation‐Associated Colorectal Cancer by Suppressing the STAT3‐CCL2 Axis and TAM‐Mediated Immunosuppression
Source: Adv Sci (Weinh). 2026 Oct 31:e78577. Online ahead of print. doi: 10.1002/advs.78577 (PMC13723711; doi:10.1002/advs.78577)
Supplement: Supplementary file 3 [file ADVS-9999-e78577-s002.docx]

**Supplemental Table 1. Docking energy table between MAGI3-PDZ1 domain**

**and STAT3 carboxy terminal TSPM sequence**

| Position in peptide TSPM | Amino acid | Amino acids interact with TSPM peptide in the PDZ1 domain | Binding free energies（kcal/mol） |
| --- | --- | --- | --- |
| 0 | Methionine, M | Phenylalanine, F | -6.0 |
| -1 | Proline, P | Threonine, T | -3.7 |
| -2 | Serine, S | Isoleucine, I | -6.0 |
| -3 | Threonine, T | Isoleucine, I | -3.6 |
