# Supplementary material for: MAGI3 Acts as an Intestinal Epithelial Gatekeeper in Inflammation‐Associated Colorectal Cancer by Suppressing the STAT3‐CCL2 Axis and TAM‐Mediated Immunosuppression
Source: Adv Sci (Weinh). 2026 Oct 31:e78577. Online ahead of print. doi: 10.1002/advs.78577 (PMC13723711; doi:10.1002/advs.78577)
Supplement: Supplementary file 4 [file ADVS-9999-e78577-s003.docx]

**Supplemental method Table 1. Histological score**

| Colonic histological features under light microscopy | score | | |
| --- | --- | --- | --- |
| Normal mucosa, no injury | | 0 |  |
| Loss of one-third of crypt glands | | 1 |  |
| Loss of two-thirds of crypt glands | | 2 |  |
| Complete loss of crypt glands, with the lamina propria covered by a single layer of epithelium and accompanied by inflammatory cell infiltration | | 3 |  |
| Mucosal epithelial erosion and destruction, with prominent inflammatory cell infiltration | | 4 |  |
