# Supplementary material for: MAGI3 Acts as an Intestinal Epithelial Gatekeeper in Inflammation‐Associated Colorectal Cancer by Suppressing the STAT3‐CCL2 Axis and TAM‐Mediated Immunosuppression
Source: Adv Sci (Weinh). 2026 Oct 31:e78577. Online ahead of print. doi: 10.1002/advs.78577 (PMC13723711; doi:10.1002/advs.78577)
Supplement: Supplementary file 5 [file ADVS-9999-e78577-s004.docx]

**Supplemental Table 4. Multivariate Cox proportional hazards regression analysis for RFS**

| **Factors** | **HR** | **95％CI** | ***P*** |
| --- | --- | --- | --- |
| Gender |  |  |  |
| Male | 1 |  |  |
| Female | 0.822 | 0.48-1.407 | 0.474 |
| Age |  |  |  |
| ＜60 | 1 |  |  |
| ≥60 | 1.038 | 0.585-1.843 | 0.898 |
| T stage |  |  |  |
| T1+T2 | 1 |  |  |
| T3+T4 | 4.574 | 1.666-12.561 | 0.003 |
| Lymph node invasion |  |  |  |
| No(N0) | 1 |  |  |
| Yes(N1/2) | 2.410 | 1.314-4.422 | 0.004 |
| Differentiation |  |  |  |
| Other | 1 |  |  |
| Good | 1.713 | 0.563-5.207 | 0.343 |
| Moderate+Poor | 0.977 | 0.457-2.09 | 0.953 |
| MAGI3 |  |  |  |
| Low | 1 |  |  |
| High | 0.369 | 0.197-0.692 | 0.002 |
|  |  |  |  |
