# Supplementary material for: MAGI3 Acts as an Intestinal Epithelial Gatekeeper in Inflammation‐Associated Colorectal Cancer by Suppressing the STAT3‐CCL2 Axis and TAM‐Mediated Immunosuppression
Source: Adv Sci (Weinh). 2026 Oct 31:e78577. Online ahead of print. doi: 10.1002/advs.78577 (PMC13723711; doi:10.1002/advs.78577)
Supplement: Supplementary file 6 [file ADVS-9999-e78577-s005.docx]

**Supplemental Table 5. Multivariate Cox proportional hazards regression analysis for RFS in the GSE39582 cohort**

| **Factors** | | **HR** | **95％CI** | ***P*** |
| --- | --- | --- | --- | --- |
| Gender | |  |  |  |
| Female | | 1 |  |  |
| Male | | 0.816 | 0.569-1.171 | 0.271 |
| Age | |  |  |  |
| ＜60 | | 1 |  |  |
| ≥60 | | 0.774 | 0.530-1.130 | 0.184 |
| T stage | |  |  |  |
| T1+T2 | | 1 |  |  |
| T3+T4 | | 4.754 | 1.502-15.043 | 0.007 |
| KRAS mutation status | |  |  |  |
| Wild-type | | 1 |  |  |
| Mutation | | 1.280 | 0.874-1.876 | 0.204 |
| BRAF mutation status | |  |  |  |
| Wild-type | | 1 |  |  |
| Mutation | | 1.304 | 0.733-2.322 | 0.365 |
| MAGI3 | |  |  |  |
| Low | | 1 |  |  |
| High | | 0.469 | 0.257-0.857 | 0.013 |
|  |  | |  |  |
