# Supplementary material for: MAGI3 Acts as an Intestinal Epithelial Gatekeeper in Inflammation‐Associated Colorectal Cancer by Suppressing the STAT3‐CCL2 Axis and TAM‐Mediated Immunosuppression
Source: Adv Sci (Weinh). 2026 Oct 31:e78577. Online ahead of print. doi: 10.1002/advs.78577 (PMC13723711; doi:10.1002/advs.78577)
Supplement: Supplementary file 7 [file ADVS-9999-e78577-s006.docx]

**Supplemental method Table 2. Primers for RT-qPCR**

| **Gene** | | **Species** | | **Sequences (5’****-3’)** |
| --- | --- | --- | --- | --- |
| *Actb* | Mouse | | F 5’-CTATGCTCTCCCTCACGCCATC-3’ | |
|  |  | | R 5’-GCCACGCTCGGTCAGGATC-3’ | |
| *Ccl2* | Mouse | | F 5’-TAAAAACCTGGATCGGAACCAAA-3’ | |
|  |  | | R 5’-GCATTAGCTTCAGATTTACGGGT-3’ | |
| *Tnf* | Mouse | | F 5’-CCTGTAGCCCACGTCGTAG-3’ | |
|  |  | | R 5’-GGGAGTAGACAAGGTACAACCC-3’ | |
| *Tgfb1* | Mouse | | F 5’-CTCCCGTGGCTTCTAGTGC-3’ | |
|  |  | | R 5’-GCCTTAGTTTGGACAGGATCTG-3’ | |
| *Arg1* | Mouse | | F 5’-AGCTCTGGGAATCTGCATGG-3’ | |
|  |  | | R 5’-ATGTACACGATGTCTTTGGCAGATA-3’ | |
| *Il10* | Mouse | | F 5’- CGGGAAGACAATAACTGCACCC-3’ | |
|  |  | | R 5’- CGGTTAGCAGTATGTTGTCCAGC-3’ | |
| *Nos2* | Mouse | | F 5’-GAGACAGGGAAGTCTGAAGCAC-3’ | |
|  |  | | R 5’-CCAGCAGTAGTTGCTCCTCTTC-3’ | |
