# Supplementary material for: MAGI3 Acts as an Intestinal Epithelial Gatekeeper in Inflammation‐Associated Colorectal Cancer by Suppressing the STAT3‐CCL2 Axis and TAM‐Mediated Immunosuppression
Source: Adv Sci (Weinh). 2026 Oct 31:e78577. Online ahead of print. doi: 10.1002/advs.78577 (PMC13723711; doi:10.1002/advs.78577)
Supplement: Supplementary file 8 [file ADVS-9999-e78577-s008.docx]

**Supplemental Table 2. Clinical and pathological characteristics of colorectal cancer patients**

| **Factors** | **Number** | **Percentage(％)** |
| --- | --- | --- |
| Gender |  |  |
| Female | 101 | 43.91 |
| Male | 129 | 56.09 |
| Age |  |  |
| ＜60 | 68 | 29.56 |
| ≥60 | 162 | 70.44 |
| T stage |  |  |
| T1+T2 | 46 | 20.00 |
| T3+T4 | 184 | 80.00 |
| Lymph node invasion |  |  |
| No(N0) | 148 | 64.35 |
| Yes(N1/2) | 82 | 35.65 |
| Differentiation |  |  |
| Good | 18 | 7.83 |
| Moderate+Poor | 184 | 80.00 |
| Other | 28 | 12.17 |
| Chemotherapy |  |  |
| Yes | 93 | 40.43 |
| No | 116 | 50.43 |
| Unclear | 21 | 9.14 |
| Recurrence |  |  |
| Non- recurrence | 161 | 70.00 |
| Recurrence | 31 | 13.48 |
| Unclear | 38 | 16.52 |
|  |  |  |
