# Supplementary material for: MAGI3 Acts as an Intestinal Epithelial Gatekeeper in Inflammation‐Associated Colorectal Cancer by Suppressing the STAT3‐CCL2 Axis and TAM‐Mediated Immunosuppression
Source: Adv Sci (Weinh). 2026 Oct 31:e78577. Online ahead of print. doi: 10.1002/advs.78577 (PMC13723711; doi:10.1002/advs.78577)
Supplement: Supplementary file 9 [file ADVS-9999-e78577-s009.docx]

**Supplemental Table 3. Univariate Cox proportional hazards regression analysis for RFS**

| **Factors** | **HR** | | **95％CI** | | ***P*** | |
| --- | --- | --- | --- | --- | --- | --- |
| Gender |  | |  | |  | |
| Male | 1 | |  | |  | |
| Female | 1.001 | | 0.593-1.688 | | 0.998 | |
| Age |  | |  | |  | |
| ＜60 | 1 | |  | |  | |
| ≥60 | 0.913 | | 0.522-1.597 | | 0.750 | |
| T stage |  | |  | |  | |
| T1+T2 | 1 | |  | |  | |
| T3+T4 | 8.809 | | 3.517-22.063 | | 3.413e-06 | |
| Lymph node invasion |  | |  | |  | |
| No(N0) | 1 | |  | |  | |
| Yes(N1/2) | 4.703 | | 2.709-8.167 | | 3.819e-08 | |
| Differentiation |  | |  | |  | |
| Other | 1 | |  | |  | |
| Good | 1.215 | | 0.421-3.502 | | 0.719 | |
| Moderate+Poor | 0.778 | | 0.366-1.654 | | 0.514 | |
| MAGI3 |  | |  | |  | |
| Low | 1 | |  | |  | |
| High | 0.308 | | 0.166-0.573 | | 0.0002 | |
|  | |  | |  | |  |
